# Projecting Global Trends and Inequalities in Adult Overweight and Obesity, 2023–2040: Findings From the NCD‐RisC Database

**DOI:** 10.1002/oby.24358

**Published:** 2025-09-02

**Authors:** Jinli Liu, Zeping Fang, Qianhui Lu, Yanan Wang, Lei Zhang

**Affiliations:** ^1^ Department of Endocrinology The First Affiliated Hospital of Xi'an Jiaotong University, Xi'an Jiaotong University Xi'an Shaanxi People's Republic of China; ^2^ China‐Australia Joint Research Center for Infectious Diseases School of Public Health, Xi'an Jiaotong University Health Science Center Xi'an Shaanxi People's Republic of China; ^3^ Key Laboratory of Environment and Genes Related to Diseases (Xi'an Jiaotong University), Ministry of Education Xi'an China; ^4^ Department of Epidemiology and Biostatistics, College of Public Health Zhengzhou University Zhengzhou Henan People's Republic of China; ^5^ Med‐X Institute, Center for Immunological and Metabolic Diseases, The First Affiliated Hospital of Xi'an Jiaotong University Xi'an Shaanxi People's Republic of China; ^6^ Phase I Clinical Trial Research Ward, The Second Affiliated Hospital of Xi'an Jiaotong University Xi'an Shaanxi People's Republic of China; ^7^ Artificial Intelligence and Modelling in Epidemiology Program, Melbourne Sexual Health Centre, Alfred Health Melbourne Australia; ^8^ School of Translational Medicine, Faculty of Medicine, Nursing and Health Sciences Monash University Melbourne Victoria Australia

**Keywords:** average annual percentage change, Bayesian age‐period‐cohort model, health inequalities, obesity, overweight, prevalence

## Abstract

**Objective:**

This study quantifies the global burden of overweight and obesity, projects future trends, and examines associated health inequalities.

**Methods:**

Overweight and obesity burden data were obtained from the NCD‐RisC database. Trends from 1990 to 2022 were analyzed, and a Bayesian model was used to project changes for 2023–2040. Cross‐national health inequalities were measured using the slope index of inequality (SII) and the relative concentration index (RCI).

**Results:**

Globally, overweight prevalence rose from 17.50% in 1990 to 28.00% in 2022 (average annual percentage change [AAPC] = 1.48%) and is projected to reach 43.97% by 2040 (AAPC = 1.13%). Obesity prevalence grew from 6.44% to 16.06% (AAPC = 2.46%) and is projected to surge to 38.96% by 2040 (AAPC = 4.96%). The SII for overweight burden dropped from 33.74% in 1990 to 16.24% in 2022, and it is projected to reverse to −2.42% by 2040. For obesity (BMI, ≥ 30.0 kg/m^2^) and mild obesity (BMI, 30.0–34.9 kg/m^2^), the SII declined from 20.12% to 18.58% and from 14.22% to 10.02%, respectively, with further drops to 9.35% and −1.70% by 2040. From 1990 to 2040, the share of the global prevalence burden of overweight and obesity in countries with the lowest GDP per capita rose from 15% to 22% and from 6% to 26%, respectively. The relative gradient inequality, measured by RCI, also showed similar findings.

**Conclusions:**

The global burden of overweight and obesity has significantly increased from 1990 to 2040. Health inequalities decreased from 1990 to 2022, with the burden mainly in higher‐income countries. However, by 2040, the burden of overweight and mild obesity is projected to shift to lower‐income countries, highlighting the need for targeted health policies and interventions.

**Study Importance**

- What is already known?
  ○ Overweight and obesity are major global health challenges, with prevalence of overweight and obesity rising significantly since 1990, particularly in high‐income countries.
  ○ Prior research lacks comprehensive global projections and systematic tracking of how the burden has shifted and is expected to shift across income‐level groups over time.
- What does this study add?
  ○ Building on prior global assessments, this study provides an extended evaluation of overweight and obesity trends across 200 countries from 1990 to 2022 and provides novel projections through 2040 using a Bayesian age‐period‐cohort model.
  ○ A systematic analysis of health inequalities further reveals that, while cross‐national disparities have narrowed overall, the burden of overweight and mild obesity is projected to shift from higher‐income to lower‐income countries.
- How might these results change the direction of research or the focus of clinical practice?
  ○ The findings underscore the urgent need for targeted policies in lower‐middle‐income countries to mitigate obesogenic drivers (e.g., urbanization, ultraprocessed food marketing) and to strengthen health systems to address the rising burden of obesity‐related chronic diseases.
  ○ Clinical and public health interventions must shift focus to prevention strategies (e.g., subsidizing healthy diets, regulating food industries) and equitable resource allocation.

## 1 Introduction

Overweight and obesity have become major public health challenges worldwide, with their prevalence increasing substantially over recent decades [1, 2]. From 1975 to 2016, global obesity prevalence tripled in men (3.2%–10.8%) and more than doubled in women (6.4%–14.9%), while the overweight rates increased markedly from 20.8% to 34.3% in men and 21.3% to 39.6% in women [1]. Regional analyses reveal stark disparities in overweight and obesity prevalence. High‐income countries, such as the United States, reported age‐standardized prevalence exceeding 75% in males and 72% in females among individuals aged 25 years and older in 2021 [3]. This increase is largely driven by shifts toward energy‐dense diets and sedentary lifestyles [4]. In contrast, low‐ and middle‐income countries (LMICs) in sub‐Saharan Africa and Southeast Asia are experiencing a concurrent rise in undernutrition and obesity—a dual burden of malnutrition [5]. In 2019, elevated BMI contributed to approximately 5 million global deaths from noncommunicable diseases (NCDs), including cardiovascular diseases, diabetes, cancers, neurological disorders, chronic respiratory diseases, and digestive disorders [6]. The economic ramifications are equally staggering: overweight‐ and obesity‐related healthcare costs and productivity losses consumed 2.19% of global GDP in 2019, and projections indicate that the economic burden will escalate to 3.29% of global GDP by 2060 if current trends persist [7]. These figures demand immediate, inequality‐focused interventions to prevent disproportionate impacts on vulnerable populations and strained healthcare systems. The NCD Global Monitoring Framework proposes that the global adult obesity rate should be maintained at or below current levels by 2025.

The World Health Organization (WHO) has positioned obesity prevention as a cornerstone of its global NCD reduction agenda. The overall goal of the WHO 25 × 25 Global Prevention Plan is to reverse the rising trend of obesity [8]. The 2022 Acceleration Plan to STOP Obesity further emphasizes systemic approaches, targeting obesogenic environments through urban planning for physical activity and subsidized access to healthy diets [9]. Despite these frameworks, implementation remains fragmented. LMICs face structural barriers, including underfunded health systems, political interference from transnational food corporations, and limited capacity to monitor policy efficacy [10, 11]. For instance, only 34% of countries have enforced WHO‐recommended food marketing regulations [12]. Compounding these challenges is the paucity of subnationally disaggregated data on obesity inequities—such as disparities across income, education, and rural–urban divides—which hinders the development of targeted interventions [13].

To date, the NCD Risk Factor Collaboration (NCD‐RisC) database stands as one of the most comprehensive and authoritative sources of global health data, particularly in the context of NCDs. This study aims to analyze the trends in adult overweight and obesity at the global, regional, and national levels from 1990 to 2022, projecting the prevalence up to 2040. Building on this analysis, the study further seeks to systematically evaluate health inequalities across socioeconomic strata, with a focus on understanding how economic status influences the distribution and burden of overweight and obesity. By synthesizing temporal trends, predictive insights, and inequality metrics, this work seeks to inform WHO‐aligned policies that prioritize marginalized populations, thereby advancing progress toward Sustainable Development Goal 3.4‐reducing premature NCD mortality by one‐third by 2030 [14].

## 2 Methods

### 2.1 Data Source

This study draws on data from multiple population‐based studies included in the NCD‐RisC database. This database compiles extensive, high‐quality data from 3663 studies, which collectively encompass 222 million participants from 200 countries and territories. These studies involved representative samples from various population groups, including adults aged 18 years and older. Height and weight measurements, which are central to the analysis of overweight and obesity, were collected from national‐, subnational‐, and community‐level data sources. The data covered a period from 1990 to 2022, with a wide geographic representation, ensuring comprehensive global coverage. The NCD‐RisC database draws from numerous sources, including nationally representative surveys such as the Demographic and Health Surveys (DHS) and Global School‐based Student Health Surveys (GSHS), as well as other health surveys and epidemiological monitoring efforts. The dataset's diversity allows for robust analysis of trends and disparities in overweight and obesity, accounting for variations across regions, income levels, and other socioeconomic factors. The collected data were analyzed using a Bayesian hierarchical meta‐regression model to estimate the prevalence and trends in overweight and obesity, with a focus on their regional and global distribution. Further details regarding the data sources, study inclusion criteria, and sampling methodology are provided in the supplementary materials of the original studies [1, 2, 15].

Socioeconomic status was defined based on the gross national per capita income, based on the World Bank classification, that is, low‐income countries (LICs), lower‐middle‐income countries (LMICs), upper‐middle‐income countries (UMICs), and high‐income countries (HICs) [16, 17]. We classified BMI into categories of overweight (25.0–29.9 kg/m^2^) and obesity (≥ 30.0 kg/m^2^), with obesity further divided into class I (mild) obesity (30.0–34.9 kg/m^2^), class II (moderate) obesity (35.0–39.9 kg/m^2^), and class III (severe) obesity (≥ 40.0 kg/m^2^) according to WHO recommendations [18].

### 2.2 Temporal Analysis of Overweight and Obesity Prevalence

In the study, the burden associated with overweight and obesity was assessed using prevalence trends over time. To analyze the trend of overweight and obesity prevalence in this study, the log‐linear model was selected. Joinpoint, a segmented regression model, was used to analyze the temporal patterns of disease distribution. A grid search method was employed to partition the study time into distinct intervals using joinpoints. This model enabled a comprehensive evaluation of temporal trends by fitting and optimizing trends within each interval. Monte Carlo permutation tests were used to ensure robustness and identify the optimal model. The annual percentage change (APC) was used to assess the trend within each independent time interval defined by the segmentation function, while the average annual percentage change (AAPC) was employed to evaluate the overall global average trend of change across multiple time intervals.

### 2.3 Bayesian Age‐Period‐Cohort (BAPC) Model for Forecasting Overweight and Obesity Burden

The study used the BAPC model to predict the prevalence of overweight and obesity. The model, based on the NCD‐RisC database covering 200 countries, incorporated probability distributions and accounted for age, period, and cohort effects. By combining data from different countries, genders, and age groups (with 5‐year intervals, where ages 18 and 19 were grouped together, and those ages 85 and above were categorized as a single group), we forecast the prevalence of overweight and obesity across regions and countries for the period from 2023 to 2040.

### 2.4 Cross‐Country Health Inequalities Analysis

We assessed the disparity in how overweight and obesity are spread across countries using two standard metrics: the slope index of inequality (SII) and the relative concentration index (RCI). These metrics helped us understand both absolute and relative gradient inequalities.

To calculate the SII, we conducted a regression analysis to evaluate the association between overweight/obesity prevalence and socioeconomic rank. A weighted regression model was used to address heteroscedasticity, and a logarithmic transformation was applied to the relative social position value to accommodate nonlinearity stemming from marginal utility. For the RCI, the cumulative relative distribution of the population by GDP per capita was compared with the rates of overweight and obesity in each country. The area under the curve was then calculated using numerical integration. If the Lorenz curve lay above the equality line, this indicated that the health burden was higher in LICs, resulting in a negative concentration index. If the Lorenz curve is below the equality line, it suggests the health burden is higher in HICs, leading to a positive concentration index.

### 2.5 Statistical Analysis

All statistical analyses were performed using the RStudio software (version 4.2.3) (https://www.r‐project.org/) and the Joinpoint Regression Program (version 4.9.0.0). A probability value of *p* < 0.05 was considered statistically significant.

## 3 Results

### 3.1 Global Trends of Overweight and Obesity

Globally, the prevalence of overweight among adults aged ≥ 18 years demonstrated a consistent upward trend, rising from 17.5% (15.84%–19.16%) in 1990 to 28.00% (24.77%–31.12%) in 2022. Projections estimate this prevalence will reach 43.97% (42.33%–45.61%) by 2040. Concurrently, AAPC of overweight prevalence increased from 1.48% (1.47%–1.49%) during 1990–2022 to 2.46% (2.42%–2.50%) for the 2023–2040 period (Table 1).

**TABLE 1:** The prevalence of overweight and obesity and AAPC during 1990–2022 and 2023–2040 at the different global levels.

Characteristics	1990		2022		2040		1990–2022	2023–2040
	Cases, million (95% CI)	Prevalence (%) (95% CI)	Cases, million (95% CI)	Prevalence (%) (95% CI)	Cases, million (95% CI)	Prevalence (%) (95% CI)	AAPC (%) (95% CI)	AAPC (%) (95% CI)
Overweight								
Global	566 (513–620)	17.5 (15.84–19.16)	1560 (1381–1734)	28.00 (24.77–31.12)	3008 (2896–3120)	43.97 (42.33–45.61)	1.48 (1.47–1.49)	2.46 (2.42–2.5)
Country income tiers								
HICs	270 (254–286)	31.33 (29.48–33.13)	361 (317–403)	32.73 (28.77–36.55)	406 (397–415)	34.27 (33.54–35)	0.14 (0.13–0.15)	0.23 (0.22–0.25)
UMICs	213 (190–235)	16.09 (14.35–17.76)	686 (621–749)	31.84 (28.84–34.76)	1151 (1071–1231)	47.77 (44.45–51.09)	2.16 (2.15–2.18)	2.18 (2.14–2.21)
LMICs	72 (61–84)	7.97 (6.71–9.27)	452 (397–506)	23.23 (20.42–25.98)	1274 (1196–1351)	48.73 (45.75–51.7)	3.39 (3.37–3.41)	4.07 (4.03–4.1)
LICs	11 (7–15)	7.69 (5.18–10.44)	61 (45–76)	16.42 (12.06–20.69)	177 (172–183)	28.03 (27.13–28.94)	2.41 (2.38–2.44)	2.96 (2.93–2.99)
Region								
Africa	24 (18–29)	9.45 (7.33–11.69)	114 (91–138)	18.06 (14.34–21.73)	278 (270–286)	26.49 (25.71–27.26)	2.04 (2.01–2.07)	2.1 (2.07–2.13)
Americas	137 (125–148)	29.61 (27.11–32.02)	262 (234–289)	34.11 (30.45–37.67)	339 (329–348)	37.87 (36.8–38.94)	0.45 (0.43–0.46)	0.49 (0.48–0.51)
Europe	205 (188–221)	33.08 (30.31–35.79)	259 (226–291)	35.10 (30.6–39.43)	289 (283–295)	37.42 (36.63–38.21)	0.19 (0.18–0.19)	0.34 (0.33–0.36)
Eastern Mediterranean	41 (34–47)	21.22 (17.82–24.61)	148 (125–170)	30.95 (26.13–35.53)	300 (286–314)	42.71 (40.74–44.68)	1.19 (1.17–1.20)	1.73 (1.7–1.76)
Southeast Asia	44 (37–51)	5.95 (5.03–6.91)	336 (302–369)	23.28 (20.93–25.59)	1015 (938–1093)	56.94 (52.6–61.28)	4.35 (4.33–4.37)	4.92 (4.89–4.96)
Western Pacific	117 (110–124)	11.93 (11.25–12.59)	440 (402–476)	29.12 (26.66–31.54)	787 (708–865)	48.03 (43.22–52.84)	2.84 (2.81–2.86)	2.72 (2.68–2.75)
Obesity								
Global	198 (175–222)	6.11 (5.42–6.87)	895 (795–1002)	16.06 (14.26–17.99)	2665 (2589–2741)	38.96 (37.85–40.08)	3.07 (3.05–3.09)	4.96 (4.89–5.03)
Country income tiers								
HICs	116 (107–125)	13.47 (12.46–14.52)	302 (272–332)	27.33 (24.65–30.10)	464 (448–481)	39.2 (37.84–40.57)	2.24 (2.22–2.25)	2.05 (2.03–2.07)
UMICs	60 (51–71)	4.55 (3.84–5.34)	349 (312–387)	16.19 (14.49–17.98)	1053 (998–1107)	43.7 (41.43–45.96)	4.05 (4.03–4.07)	5.53 (5.47–5.58)
LMICs	19 (15–23)	2.06 (1.69–2.49)	213 (186–243)	10.95 (9.57–12.46)	948 (898–998)	36.26 (34.35–38.17)	5.36 (5.34–5.38)	6.72 (6.67–6.76)
LICs	3 (2–4)	1.87 (1.25–2.74)	32 (24–40)	8.60 (6.60–10.96)	200 (193–208)	31.65 (30.47–32.83)	4.88 (4.80–4.96)	7.35 (7.28–7.42)
Region								
Africa	8 (6–10)	3.14 (2.49–3.92)	68 (57–81)	10.75 (8.94–12.80)	293 (284–303)	27.94 (27.05–28.83)	3.91 (3.87–3.96)	5.35 (5.29–5.42)
Americas	62 (56–68)	13.35 (12.13–14.67)	264 (244–285)	34.41 (31.77–37.13)	548 (528–569)	61.29 (59.03–63.56)	3.00 (2.98–3.02)	3.16 (3.14–3.19)
Europe	90 (80–100)	14.48 (12.97–16.1)	189 (167–212)	25.57 (22.56–28.69)	275 (269–281)	35.61 (34.82–36.4)	1.80 (1.78–1.82)	1.86 (1.83–1.89)
Eastern Mediterranean	17 (14–20)	8.97 (7.51–10.61)	131 (116–147)	27.39 (24.3–30.7)	450 (427–472)	64.04 (60.78–67.3)	3.55 (3.54–3.56)	4.73 (4.68–4.78)
Southeast Asia	7 (5–9)	0.95 (0.75–1.20)	116 (101–132)	8.03 (6.98–9.18)	585 (538–631)	32.78 (30.19–35.36)	6.89 (6.86–6.92)	7.89 (7.86–7.93)
Western Pacific	15 (13–16)	1.48 (1.34–1.64)	127 (110–145)	8.39 (7.30–9.58)	514 (463–565)	31.4 (28.28–34.51)	5.58 (5.55–5.61)	7.34 (7.28–7.4)

Abbreviations: AAPC, average annual percentage change; HICs, high‐income countries; LICs, low‐income countries; LMICs, lower‐middle‐income countries; UMICs, upper‐middle‐income countries.

**FIGURE 1:**
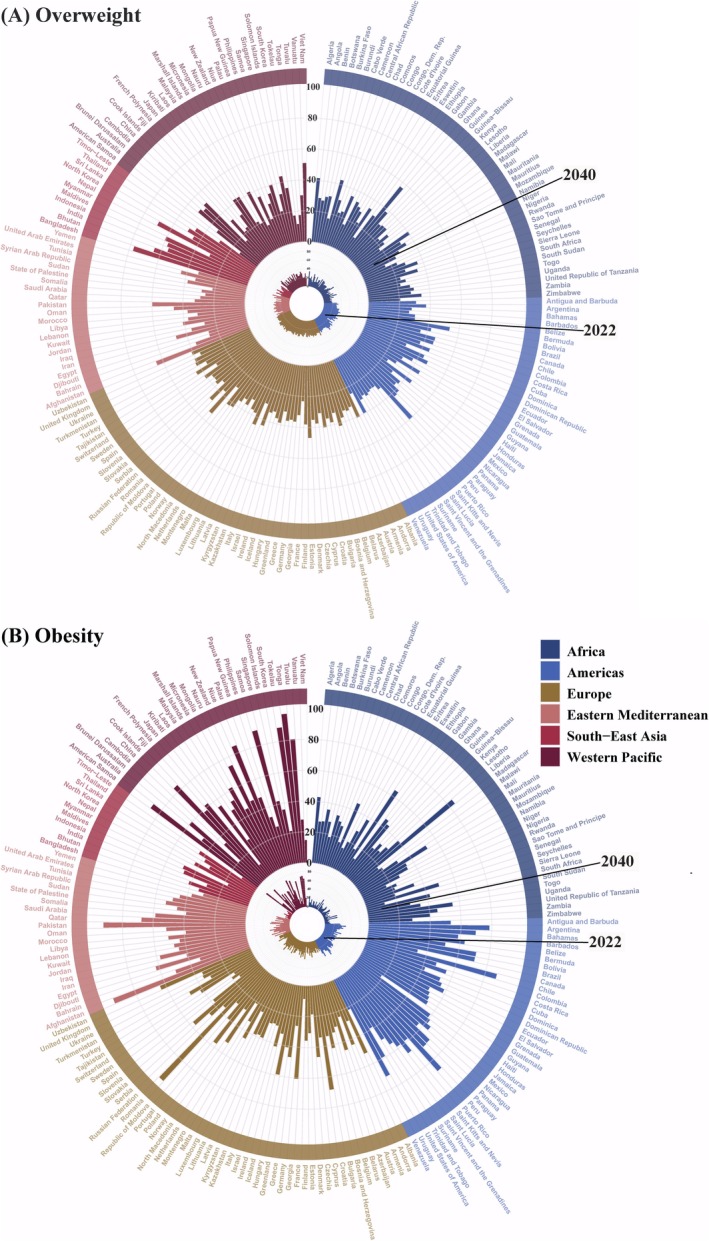
Prevalence of (A) overweight and (B) obesity by country and region in 2022 and 2040 among adults (age ≥ 18 years). [Color figure can be viewed at wileyonlinelibrary.com]

Similarly, obesity prevalence showed sustained growth from 1990 to 2040 (Figure 1). The global obesity rate increased from 6.11% (5.42%–6.87%) in 1990 to 16.06% (14.26%–17.99%) in 2022, with AAPC of 3.07% (3.05%–3.09%) (Table 1). Projections estimate this prevalence will reach 38.96% (37.85%–40.08%) by 2040, with AAPC of 4.96% (4.89%–5.03%) during 2023–2040 (Table 1).

### 3.2 Global Trends of Overweight and Obesity by Income Tiers

Overweight prevalence exhibited universal growth across national income categories from 1990 to 2040, with distinct acceleration patterns. LMICs demonstrated the most pronounced escalation, rising from 7.97% (6.71%–9.27%) in 1990 to 23.23% (20.42%–25.98%) by 2022 (AAPC = 3.39% [3.37%–4.41%], 1990–2022) and projected to reach 48.73% (45.75%–51.70%) in 2040 at an accelerated AAPC of 4.07% (4.03%–4.10%) for 2023–2040. Conversely, HICs displayed minimal growth, advancing from 31.33% (29.48%–33.13%) to 32.73% (28.77%–36.55%) in 2022 (AAPC = 0.14% [0.13%–0.15%]), with projections suggesting marginal progression to 34.27% (33.54%–35.00%) by 2040 (AAPC = 0.23% [0.22%–0.25%]) (Table 1).

A parallel but more acute trajectory characterized global obesity patterns during 1990–2040. Notably, LMICs exhibited the most dramatic surge, with prevalence climbing 5.3‐fold from 2.06% (1.69%–2.49%) in 1990 to 10.95% (9.57%–12.46%) in 2022 (AAPC = 5.36% [5.34%–5.38%]) and projected to quintuple further to 36.26% (34.35%–38.17%) by 2040 (AAPC = 6.72% [6.67%–6.76%]). While HICs maintained lower growth rates, their baseline‐adjusted increase remained significant: from 13.47% (12.46%–14.52%) to 27.33% (24.65%–30.10%) in 2022 (AAPC = 2.24% [2.22%–2.25%]), with projected progression to 39.2% (37.84%–40.57%) by 2040 despite deceleration to AAPC = 2.05% (2.03%–2.07%) (Table 1).

### 3.3 Global Trends of Overweight and Obesity by Geographical Regions

**FIGURE 2:**
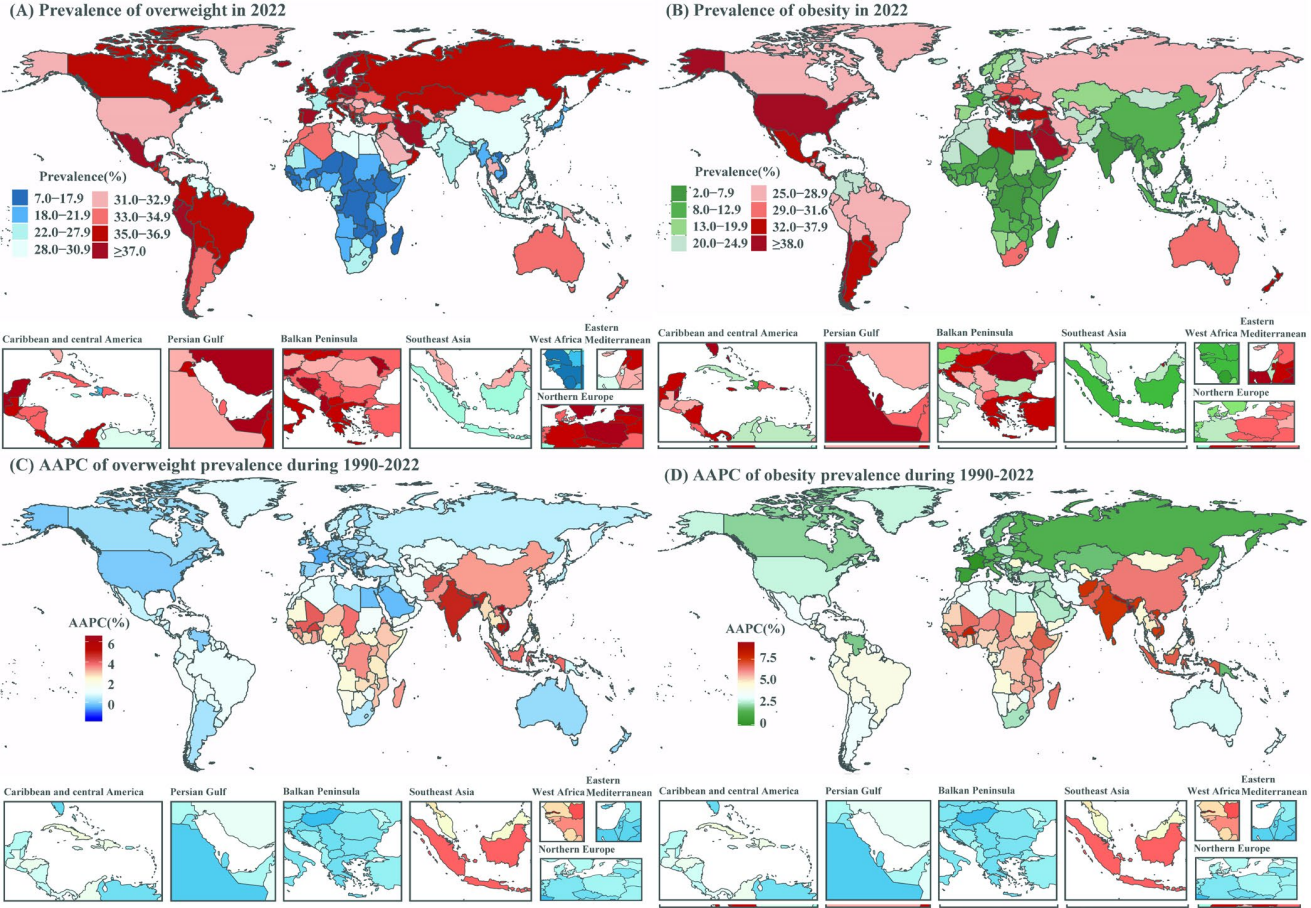
Global distribution of overweight prevalence in (A) 2022 and (B) 2040, with corresponding average annual percentage change (AAPC) (C) from 1990 to 2022 and (D) from 2023 to 2040 by country and territory. [Color figure can be viewed at wileyonlinelibrary.com]

From 1990 to 2040, the prevalence of overweight and obesity increased steadily across all regions, although the rates varied significantly (Table 1). In 1990, Europe and the Western Pacific had relatively high rates of overweight, approaching or exceeding 30%, while Africa and Southeast Asia had lower rates, generally below 10% (Figure S1A). By 2022, 5 countries (2.5%) had overweight prevalence exceeding 40%, primarily in the Americas and Europe (Table S1; Figure 1A; Figure 2A), while 35 countries (18%) had prevalence below 20%, mostly in sub‐Saharan Africa (Table S1; Figure 1A; Figure 2A). Sub‐Saharan Africa, South Asia, and Southeast Asia experienced higher AAPC in overweight prevalence from 1990 to 2022, reflecting faster growth in these regions (Figure 2C). Projections for 2040 suggest that the number of countries with overweight prevalence exceeding 40% will rise significantly, reaching 55 (27%), predominantly in South America, Europe, South Asia, and Southeast Asia (Table S1; Figure 2B). Conversely, the number of countries with prevalence below 20% is expected to decrease to 13 (6.5%), mainly in Africa and the Eastern Mediterranean (Table S1; Figure 2B). Sub‐Saharan Africa, South Asia, and Southeast Asia are projected to experience significantly higher AAPC in overweight prevalence from 2023 to 2040, indicating rapid growth in these regions (Figure 2D).

**FIGURE 3:**
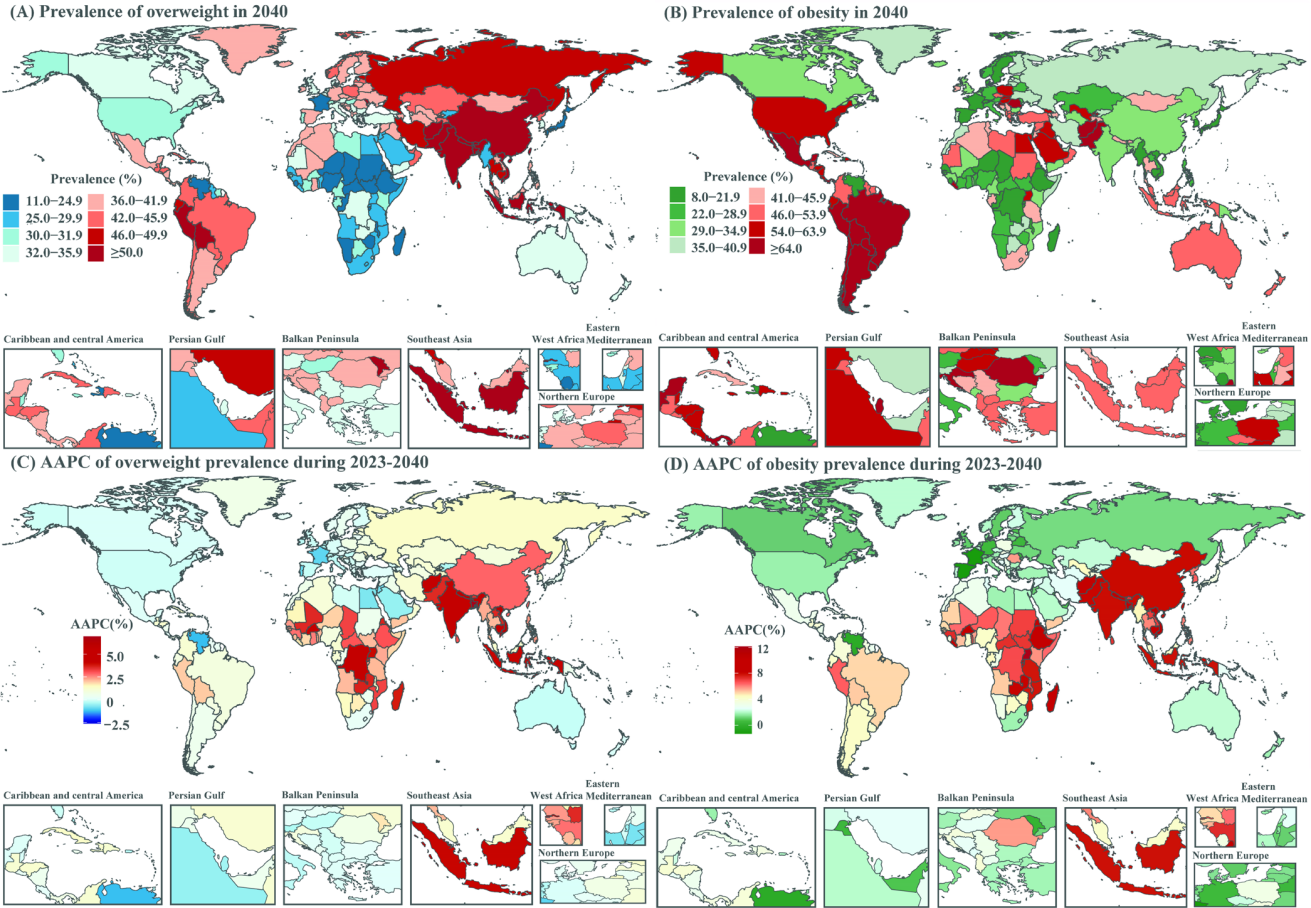
Global distribution of obesity prevalence in (A) 2022 and (B) 2040, with corresponding average annual percentage change (AAPC) (C) from 1990 to 2022 and (D) from 2023 to 2040 by country and territory. [Color figure can be viewed at wileyonlinelibrary.com]

In 1990, obesity prevalence was higher in the Western Pacific, approaching or exceeding 40%, while Africa and Southeast Asia had lower rates, generally below 10% (Figure S1B). By 2022, 22 countries (11%) had obesity prevalence exceeding 40%, mostly in the Americas, Eastern Mediterranean, and Western Pacific (Table S2; Figure 1B; Figure 3A), while 33 countries (17%) had prevalence below 10%, mainly in sub‐Saharan Africa and the Western Pacific (Table S2; Figure 1B; Figure 3A). From 1990 to 2022, sub‐Saharan Africa, South Asia, and Southeast Asia saw higher AAPC in obesity prevalence, indicating faster growth (Figure 3C). Projections for 2040 suggest that the number of countries with obesity prevalence exceeding 40% is projected to increase more than fivefold, reaching 108 (54%), mainly in the Americas, Eastern Mediterranean, and North Africa (Table S2; Figure 3B). Meanwhile, countries with obesity prevalence below 20% are expected to decrease to 20 (10%), primarily in Africa (Figure 1B; Figure 3B). Sub‐Saharan Africa, South Asia, and Southeast Asia are projected to experience significantly higher AAPC in obesity prevalence from 2023 to 2040, indicating rapid growth in these regions (Figure 3D).

### 3.4 Global Health Inequalities of Overweight and Obesity

**FIGURE 4:**
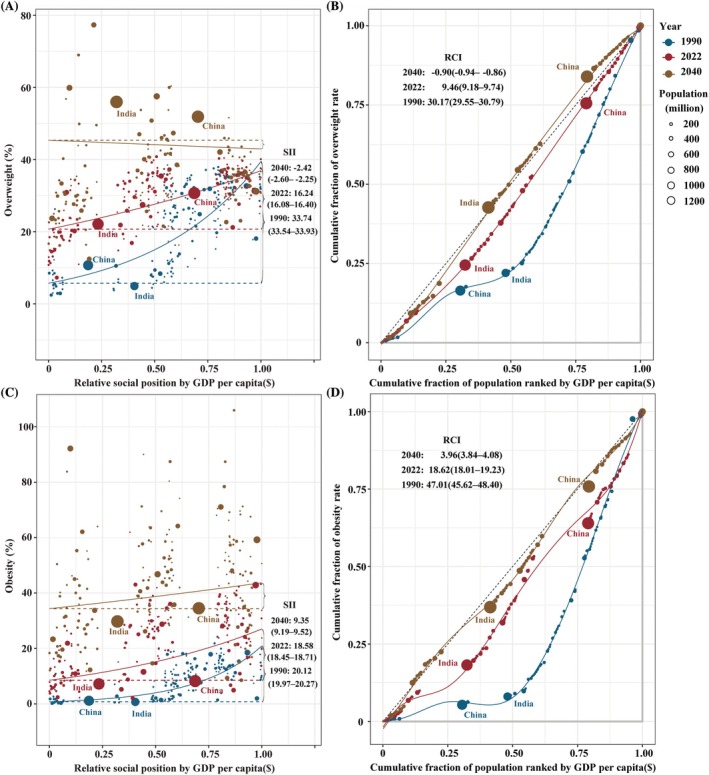
Income‐related health inequality analysis for overweight and obesity prevalence by country or territory (1990, 2022, 2040). (A, C) Absolute inequality: GDP per capita‐related regression curves with slope index of inequality (SII, 95% CI). (B, D) Relative inequality: GDP per capita‐related concentration curves with relative concentration index (RCI, 95% CI). [Color figure can be viewed at wileyonlinelibrary.com]

Our analysis revealed significant disparities in the prevalence of overweight and obesity, measured through both absolute and relative gradient inequalities. The SII for global overweight prevalence reflected a disparity of 33.74% (33.54% to 33.93%) between countries with the lowest and highest GDP per capita in 1990 (Table 2). The positive sign of the SII suggested a disproportionate concentration of the overweight burden in HICs during this period. By 2022, this absolute inequality had decreased to 16.24% (16.08% to 16.40%), and it is projected to further decline to −2.42% (−2.60% to −2.25%) by 2040 (Table 2). The negative SII value by 2040 indicates a shift in the overweight burden, with LICs expected to bear a disproportionate share relative to HICs. Similarly, the RCI for overweight prevalence decreased from 30.17% (29.55% to 30.79%) in 1990 to 9.46% (9.18% to 9.74%) in 2022 and is projected to reach −0.90% (−0.94% to −0.86%) by 2040 (Table 2). These trends demonstrate that overweight prevalence, which was historically more concentrated in wealthier countries (1990–2022), is expected to shift toward LICs by 2040 (Table 2; Figure 4A,B). In 1990 and 2022, countries in the lowest quartile of GDP per capita accounted for approximately 15% and 18% of the global prevalence burden associated with overweight, while this proportion increased to 22% by 2040 (Figure 4B).

**TABLE 2:** The health inequality regression and concentration curves for the prevalence related to overweight and obesity during 1990–2022 and 2040.

Characteristics (BMI)	1990	2022	2040
Overweight (25.0–29.9 kg/m^2^)			
SII (%, 95% CI)	33.74 (33.54–33.93)	16.24 (16.08–16.40)	−2.42 (−2.60 to −2.25)
RCI (%, 95% CI)	30.17 (29.55–30.79)	9.46 (9.18–9.74)	−0.90 (−0.94 to −0.86)
Obesity (≥ 30.0 kg/m^2^)			
SII (%, 95% CI)	20.12 (19.97–20.27)	18.58 (18.45–18.71)	9.35 (9.19–9.52)
RCI (%, 95% CI)	47.01 (45.62–48.40)	18.62 (18.01–19.23)	3.96 (3.84–4.08)
Class I (mild) obesity (30.0–34.9 kg/m^2^)			
SII (%, 95% CI)	14.22 (14.22–14.23)	10.02 (10.00–10.04)	−1.70 (−1.71 to −1.70)
RCI (%, 95% CI)	45.45 (43.79–47.11)	15.06 (14.38–15.73)	−1.16 (−1.20 to −1.12)
Class II (moderate) obesity (35.0–39.9 kg/m^2^)			
SII (%, 95% CI)	4.07 (4.06–4.08)	4.80 (4.78–4.82)	3.36 (3.36–3.36)
RCI (%, 95% CI)	50.88 (47.71–54.05)	22.01 (20.57–23.45)	5.20 (4.96–5.45)
Class III (severe) obesity (≥ 40.0 kg/m^2^)			
SII (%, 95% CI)	1.46 (1.44–1.48)	3.53 (3.53–3.53)	10.69 (10.69–10.69)
RCI (%, 95% CI)	52.38 (47.5–57.25)	30.68 (27.39–33.98)	23.76 (19.93–27.59)

Abbreviations: RCI, relative concentration index; SII, slope index of inequality.

Contrasting this pattern, obesity inequalities persisted with a stable concentration in wealthier countries. The SII for global obesity prevalence demonstrated a disparity of 20.12% (19.97%–20.27%) between countries with the lowest and highest GDP per capita in 1990 (Table 2). This absolute inequality widened to 18.58% (18.45%–18.71%) by 2022 and is projected to drop further to 9.35% (9.19%–9.52%) by 2040 (Table 2). Similarly, the RCI for relative inequality fell sharply from 47.01% (45.62%–48.40%) in 1990 to 3.96% (3.84%–4.08%) by 2040, showing obesity remains more common in wealthier populations but has become less concentrated over time (Table 2; Figure 4C,D). In 1990 and 2022, the countries in the lower quartile of GDP per capita carried approximately 6% and 11% of the global prevalence burden of obesity, while this figure rose to 26% in 2040 (Figure 4D).

Obesity was further divided into class I (mild) obesity (30.0–34.9 kg/m^2^), class II (moderate) obesity (35.0–39.9 kg/m^2^), and class III (severe) obesity (≥ 40.0 kg/m^2^). The SII for mild (I) obesity prevalence showed disparities ranging from 14.22% (14.22% to 14.23%) in 1990 to 10.02% (10.00% to 10.04%) by 2022, with projections indicating a further decline to −1.70% (−1.71% to −1.70%) by 2040 (Table 2).

## 4 Discussion

Our study reports a consistent increase in both overweight and obesity prevalence among adults aged ≥ 18 years from 1990 to 2040. Specifically, overweight is projected to rise from 28.00% in 2022 to 43.97% by 2040, while obesity is expected to grow more rapidly, from 16.06% in 2022 to 38.96% by 2040, consistent with previous findings [2, 3]. Over this period, global overweight and obesity prevalence showed significant growth across all income tiers, with the most pronounced increases observed in low‐ and middle‐income countries, while growth in high‐income countries remained minimal. From 1990 to 2040, both overweight and obesity rates rose across all regions, with regions such as sub‐Saharan Africa, South Asia, and Southeast Asia showing the fastest growth. The burden of overweight and obesity remained mainly concentrated in higher‐income countries between 1990 and 2022. However, by 2040, it is projected that the burden of overweight and mild obesity will shift predominantly to lower‐income countries.

The study highlights a significant increase in overweight and obesity prevalence among adults aged 18 and above from 1990 to 2040, with obesity increasing more rapidly than overweight. This trend presents an important area of inquiry. The advice to simply “eat less and exercise more” for weight loss oversimplifies the complex physiological interactions and environmental influences underlying obesity [19, 20, 21]. This method may have some value in certain cases, but it fails to consider the multifaceted challenges of overweight and obesity as a global epidemic. The causes of overweight and obesity extend beyond personal habits, involving a complex interplay of biological, social, environmental, and economic influences. Genetic, epigenetic, and neuroendocrine factors play a role in determining how the body handles fat storage and burning, appetite control, and energy expenditure [22, 23]. In addition to the biological aspects, it is crucial to address the social, environmental, and economic drivers of overweight and obesity. The increased availability and marketing of calorie‐dense [19], highly processed foods [21], along with the sedentary nature of many jobs and daily activities, contribute significantly to the rise in overweight and obesity prevalence [20]. In China, for example, the shift from traditional diets rich in vegetables and grains to energy‐dense, processed foods has been accompanied by an increase in overweight and obesity prevalence [24, 25]. Similarly, societal development has led to more intellectual and office‐based job opportunities, resulting in a significant decline in physical activity [26]. The widespread availability and easy access to unhealthy food options, such as takeaway meals and ultraprocessed foods in supermarkets, have contributed to the worsening global obesity crisis [27]. One key factor for obesity's faster increase than overweight is the accumulation of risk factors related to its pathophysiology. Biologically, once overweight, an individual is more likely to develop obesity because of physiological mechanisms (e.g., leptin resistance, altered neuroendocrine signaling) promoting fat storage [22, 23]. This feedback loop exacerbates weight gain and contributes to a growing obesity burden. As the obesity problem intensifies, its economic burden is also growing. In 2019, the economic impact of overweight and obesity was estimated to account for 2.19% of global GDP [7].

The rapid rise in overweight and obesity rates across all regions from 1990 to 2040, particularly in sub‐Saharan Africa, South Asia, and Southeast Asia, is striking. Historically, these regions were linked to undernutrition, but their swift shift toward obesity highlights the global impact of dietary changes and urbanization. In Southeast Asia, the growing obesity trend underscores the influence of multinational food companies in reshaping food environments [28]. For example, the widespread expansion of Western‐style fast‐food chains and sugary beverages in urban areas of countries like Malaysia and the Philippines has made diets high in refined carbs, saturated fats, and sugars more common, replacing traditional plant‐based, fiber‐rich diets [28]. The pace of obesity growth in these regions is notably faster than historical trends observed in Western countries, pointing to distinct vulnerabilities. Sub‐Saharan Africa has fragmented health systems that are inadequately equipped to implement effective obesity prevention strategies. By 2023, only a few countries in the region have implemented sugar‐sweetened beverage taxes, and these policies have yet to achieve widespread impact [29]. Meanwhile, South Asia's agricultural policies continue to prioritize calorie‐dense crops like rice and wheat, while largely neglecting pulses and vegetables, which inadvertently fosters obesogenic diets [30]. Without effective interventions, these regions may experience a substantial rise in obesity‐related chronic diseases, further widening health disparities.

Obesity remains more prevalent in high‐income countries. In the United States, obesity rates plateaued at 42% by 2022 after decades of increase, partly due to public health initiatives targeting sugar‐sweetened beverages and trans fats [1]. In contrast, overweight and obesity rates in India have been rising steadily—among males from 37.71% in 2015–2016 to 44.02% in 2019–2021 and among females from 36.14% to 41.16%, with increased consumption of snacks playing a contributing role [31, 32]. This urban migration has facilitated reliance on energy‐dense, processed foods. The global consumption of ultraprocessed foods continues to rise, with the highest consumption in high‐income countries, although the growth rate is slower (0.4% annual growth rate from 2009 to 2019) [33]. In contrast, the growth rate is faster in low‐ and middle‐income countries (2.8% and 4.4%, respectively), especially in Asia, the Middle East, and Africa. This trend is closely linked to the increasing prevalence of overweight and obesity [33].

Our study assessed the inequalities in overweight and obesity by GDP per capita. Our findings indicate that the obesity burden remained disproportionately concentrated in higher‐income countries, and the overweight burden showed a notable shift from being more prevalent in higher‐income countries to becoming more widespread in lower‐income countries. In higher‐income countries, obesity rates have plateaued in recent years, partly due to public health interventions such as taxes on sugar‐sweetened beverages, improved dietary guidelines, and widespread awareness campaigns [34]. However, these interventions have not been equally successful in lower‐income countries. Economic growth boosts access to calorie‐dense products like ultraprocessed foods and sugary beverages, often replacing traditional diets high in fruits, vegetables, and whole grains in developing countries [35]. One of the key drivers of the significant shift in the burden of overweight from high‐income to low‐income countries is rapid urbanization and the globalization of unhealthy diets, particularly the increased consumption of ultraprocessed foods and sugary beverages, which are often aggressively marketed in developing countries. Classifying obesity into mild (class I), moderate (class II), and severe (class III) provides a clearer understanding of the issue. From 1990 to 2022, mild obesity was more common in high‐income countries. However, by 2024, this trend is expected to shift toward lower‐income countries. This change indicates that mild obesity is gradually spreading to lower‐income countries. In the future, moderate and severe obesity may also follow this trend. Additionally, this growing burden is likely to increase chronic diseases related to obesity, putting more pressure on healthcare systems, particularly in low‐ and middle‐income countries where resources are already scarce [36].

Our study has several limitations. First, a few low‐ and middle‐income countries, particularly in sub‐Saharan Africa and parts of South Asia, lack reliable data on overweight and obesity prevalence. The estimates for these countries are largely based on geographic hierarchical models, using data from other countries, which can affect the accuracy and completeness of the results for these regions. We have conducted a systematic assessment of the data inferred from neighboring countries and have carefully considered the confidence intervals (CI) to ensure the robustness of the data. Second, in this study, the thresholds for overweight and obesity across 200 countries were defined based on WHO standards. However, using uniform BMI cutoff points may not fully reflect the differences in obesity‐related health risks among diverse ethnic groups. BMI does not take into account variations in fat distribution and body composition, which may lead to biased assessments of obesity status and associated health risks in certain populations. Third, the concentration index employed in this study can be influenced by countries with large populations. To address this, future analyses should delve into subnational data, allowing for a more balanced consideration of variations in population size among countries.

In conclusion, the global burden of overweight and obesity has significantly increased from 1990 to 2040, with a projected acceleration in prevalence between 2023 and 2040. The most significant growth is observed in low‐ and middle‐income countries, with minimal increases in high‐income countries. Regions such as sub‐Saharan Africa, South Asia, and Southeast Asia are expected to experience the fastest growth. From 2022 to 2040, the burden of overweight has notably shifted from higher‐income to lower‐income countries. Obesity remains more concentrated in higher‐income countries, but the prevalence of mild obesity is expected to shift toward lower‐income countries by 2024. To address this rising global health challenge, coordinated efforts are needed to curb obesity trends and reduce health disparities across income groups.

## Author Contributions

L.Z. and J.L. substantially contributed by developing the conceptual framework and design of the study. J.L. wrote the first draft of the manuscript and performed the statistical analysis. Z.F. and Q.L. have accessed and verified the data. L.Z., Z.F., Q.L., and Y.W. critically revised the manuscript for important intellectual content. L.Z. was responsible for the decision to submit the manuscript. All authors confirm that they had full access to all the data in the study and approved the final version for publication.

## Conflicts of Interest

The authors declare no conflicts of interest.

## Supporting information


Data S1.

## Data Availability

The data that support the findings of this study are available in the NCD Risk Factor Collaboration (NCD‐RisC) at https://www.ncdrisc.org/index.html. These data were derived from the following resources available in the public domain: 10.1016/S0140‐6736(23)02750‐2, https://www.ncdrisc.org/index.html.
