# Supplementary material for: Projecting Global Trends and Inequalities in Adult Overweight and Obesity, 2023–2040: Findings From the NCD‐RisC Database
Source: Obesity (Silver Spring). 2025 Sep 2;33(10):1955–67. doi: 10.1002/oby.24358 (PMC12477102; doi:10.1002/oby.24358)
Supplement: Supplementary file 1 — Data S1. [file OBY-33-1955-s001.pdf]

Supplemental Materials：

Projecting global trends and inequalities in adult overweight and obesity, 2023-2040: Findings from the NCD-RisC database

This appendix provided more methodological detail on spatial autocorrelation analysis and relative results.

Contents

1. Supplementary Figures……………………………………………………………………………………………2

Figure S1. Prevalence of overweight(A) and obesity(B) by country and region in 1990 (A) among adults (age ≥18 years) …………………………2

2. Supplementary Tables……………………………………………………………………………………………3

Table S1. The prevalence of overweight and average annual percentage change during 1990–2040 by geographical region and countries…………3

Table S2. The prevalence of obesity and average annual percentage change during 1990–2040 by geographical region and countries……………12

1. Supplementary Figures

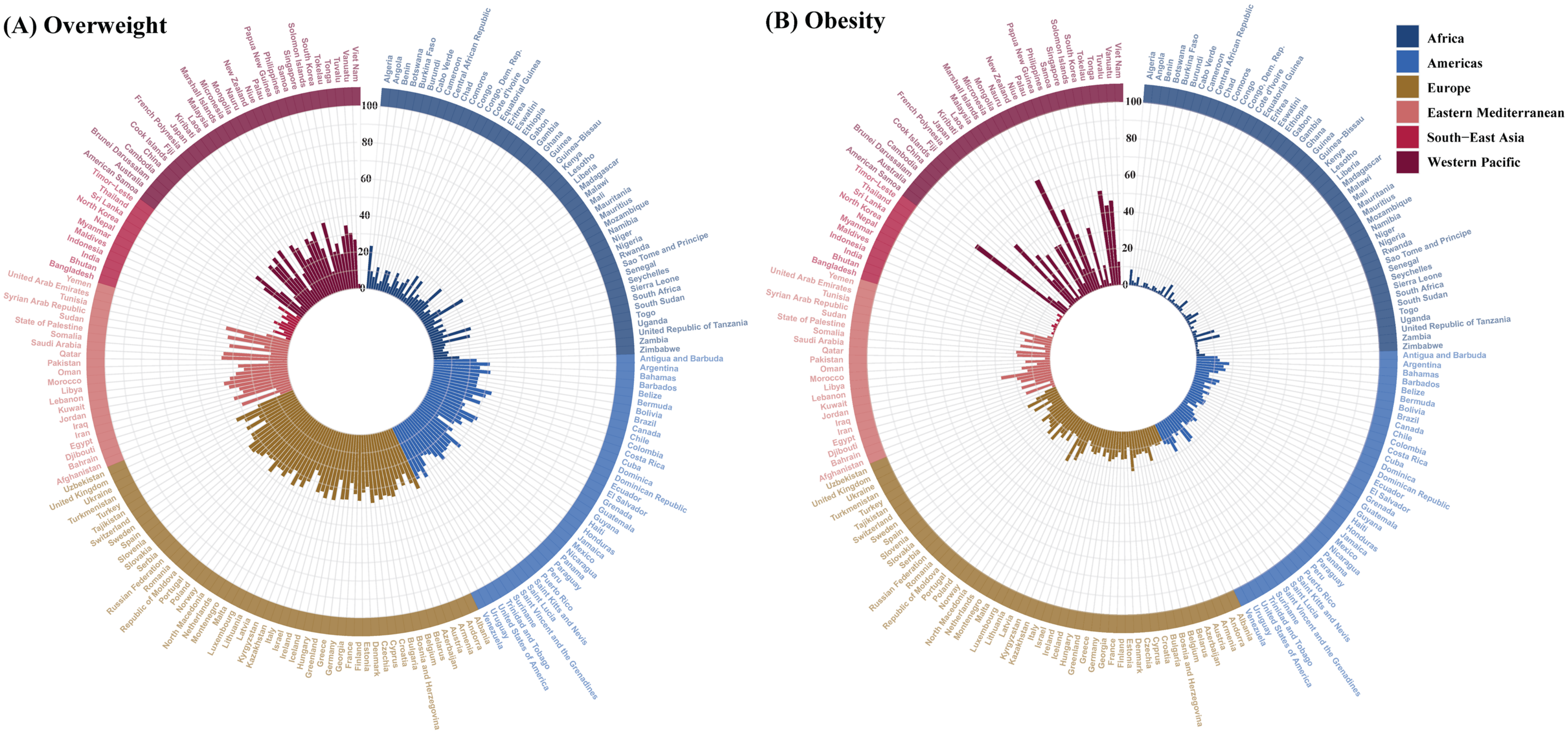

Figure S1. Prevalence of overweight (A) and obesity (B) by country and region in 1990 among adults (age ≥18 years)

2. Supplementary Tables

Table S1. The prevalence of overweight and average annual percentage change (AAPCs) during 1990–2040 by geographical regions and countries

Characteristics	1990		2022		2040		1990-2022	2023-2040
	Cases, NO, ×10^3(95% CI)	Prevalence (%) No. (95% CI)	Cases, NO, ×10^3(95% CI)	Prevalence (%) No. (95% CI)	Cases, NO, ×10^3(95% CI)	Prevalence (%) No. (95% CI)	AAPC (%) No. (95% CI)	AAPC (%) No. (95% CI)
**Africa**								
Algeria	3051(2387–3683)	23.54(18.42–28.42)	10061(8531–11518)	34.25(29.04–39.21)	16846(15681–18011)	41.45(38.59–44.32)	1.18(1.17–1.19)	1.02(0.98–1.06)
Angola	558(286–831)	9.81(5.03–14.62)	3341(2003–4650)	19.23(11.53–26.77)	9157(8215–10099)	28.7(25.75–31.65)	2.13(2.1–2.15)	2.12(2.08–2.16)
Benin	171(123–226)	6.81(4.9–9.03)	1279(1010–1541)	18.11(14.3–21.82)	3232(2794–3670)	27.34(23.63–31.05)	3.1(3.06–3.14)	2.16(2.13–2.19)
Botswana	78(50–108)	12.44(7.9–17.17)	341(249–428)	22.75(16.61–28.56)	637(578–696)	30.07(27.29–32.86)	1.9(1.88–1.92)	1.66(1.66–1.66)
Burkina Faso	161(116–213)	3.76(2.72–4.99)	1647(1404–1896)	14.61(12.45–16.82)	5999(5243–6755)	30.95(27.05–34.85)	4.34(4.29–4.39)	4.11(4.09–4.13)
Burundi	134(69–214)	5.35(2.76–8.51)	674(314–1045)	10.75(5–16.67)	1916(1715–2118)	16.68(14.93–18.44)	2.21(2.19–2.23)	2.41(2.37–2.44)
Cabo Verde	21(14–28)	11.88(8.05–15.76)	105(91–118)	29.97(26.09–33.67)	192(174–211)	42.91(38.8–47.02)	2.93(2.91–2.95)	1.9(1.88–1.92)
Cameroon	626(474–780)	11.69(8.85–14.56)	3365(2751–3960)	23.74(19.41–27.94)	7359(6488–8230)	30.15(26.58–33.72)	2.23(2.18–2.28)	1.2(1.19–1.22)
Central African Republic	78(49–110)	5.45(3.48–7.74)	281(178–385)	12.8(8.12–17.53)	921(819–1022)	22.25(19.8–24.69)	2.69(2.66–2.73)	3.02(3.01–3.04)
Chad	112(67–165)	4.05(2.42–6)	1108(674–1540)	13.11(7.98–18.23)	3794(3341–4247)	23.73(20.89–26.56)	3.75(3.71–3.8)	3.23(3.21–3.26)
Comoros	26(19–33)	12.53(9.13–16.16)	121(93–149)	25.88(19.8–31.77)	248(225–270)	35.34(32.19–38.5)	2.3(2.26–2.34)	1.73(1.7–1.75)
Congo	98(78–120)	8.43(6.7–10.28)	466(314–615)	14.75(9.93–19.47)	872(783–961)	16.4(14.72–18.07)	1.78(1.73–1.82)	0.52(0.5–0.53)
Cote d'Ivoire	501(370–640)	9.03(6.66–11.53)	3365(2567–4153)	21.32(16.26–26.31)	8269(7315–9222)	30.9(27.34–34.46)	2.72(2.69–2.76)	1.85(1.8–1.9)
Democratic Republic of the Congo	880(570–1239)	4.85(3.14–6.83)	7026(5136–8926)	14.46(10.57–18.37)	29821(25929–33713)	33.37(29.01–37.72)	3.47(3.44–3.51)	4.58(4.54–4.62)
Equatorial Guinea	30(17–44)	12.1(6.75–17.6)	219(125–312)	21.42(12.23–30.56)	465(420–511)	28.67(25.88–31.46)	1.8(1.78–1.83)	1.56(1.53–1.58)
Eritrea	38(26–54)	3.84(2.57–5.38)	207(130–289)	11.39(7.17–15.91)	639(580–698)	21.69(19.68–23.7)	3.46(3.44–3.48)	3.65(3.64–3.67)
Eswatini	65(43–86)	16.25(10.71–21.72)	194(150–237)	26.69(20.65–32.59)	324(294–354)	33.79(30.67–36.9)	1.56(1.54–1.58)	1.3(1.28–1.33)
Ethiopia	661(432–952)	2.92(1.91–4.21)	4871(3706–6115)	7.28(5.54–9.14)	14137(12816–15459)	12.47(11.31–13.64)	2.9(2.85–2.95)	3.07(3.06–3.07)
Gabon	77(54–100)	15.12(10.59–19.74)	354(260–441)	25.26(18.57–31.5)	681(613–748)	31.33(28.21–34.46)	1.62(1.59–1.64)	1.1(1.07–1.13)

Gambia	28(19–37)	5.42(3.82–7.17)		341(268–412)	24.87(19.53–30.03)		1310(1003–1617)	56.82(43.52–70.12)		4.89(4.84–4.94)	4.45(4.39–4.51)
Ghana	613(468–767)	8.29(6.33–10.37)		4439(3808–5059)	23.41(20.08–26.68)		10901(9471–12331)	38.26(33.24–43.28)		3.29(3.26–3.33)	2.63(2.59–2.67)
Guinea	195(133–266)	6.01(4.11–8.21)		1285(946–1628)	17.67(13.01–22.38)		3500(3118–3883)	29.34(26.13–32.55)		3.44(3.4–3.47)	2.75(2.73–2.77)
Guinea-Bissau	35(20–51)	7.48(4.22–11.02)		223(133–309)	19.72(11.74–27.38)		554(491–617)	30.26(26.83–33.69)		3.08(3.06–3.1)	2.32(2.3–2.34)
Kenya	792(609–993)	7.92(6.09–9.93)		6047(5294–6789)	20.3(17.77–22.79)		14091(12815–15366)	30.39(27.64–33.14)		2.99(2.97–3)	2.26(2.24–2.28)
Lesotho	176(144–209)	19.14(15.64–22.79)		270(201–338)	20.22(15.02–25.29)		409(375–443)	22.32(20.47–24.18)		0.17(0.17–0.18)	0.62(0.6–0.64)
Liberia	90(60–124)	8.18(5.41–11.21)		573(490–655)	20.32(17.38–23.21)		1313(1159–1467)	28.32(25–31.65)		2.88(2.85–2.9)	1.75(1.73–1.78)
Madagascar	238(162–324)	4.17(2.85–5.68)		1942(1291–2617)	11.91(7.92–16.05)		6211(5671–6751)	23.26(21.24–25.28)		3.34(3.32–3.37)	3.77(3.75–3.79)
Malawi	335(251–427)	7.25(5.42–9.24)		1543(1233–1839)	14.78(11.81–17.61)		3444(3117–3771)	18.44(16.69–20.19)		2.25(2.23–2.28)	1.31(1.3–1.32)
Mali	254(177–338)	5.93(4.15–7.91)		2195(1650–2708)	20.57(15.46–25.38)		7905(6724–9087)	40.26(34.24–46.28)		3.97(3.93–4.01)	3.58(3.55–3.62)
Mauritania	111(77–147)	11.92(8.28–15.75)		576(427–728)	23.65(17.54–29.88)		1386(1217–1556)	32.06(28.14–35.98)		2.16(2.13–2.19)	1.58(1.55–1.61)
Mauritius	178(164–191)	25.23(23.25–27.2)		338(283–393)	32.97(27.62–38.34)		351(323–378)	34.67(31.98–37.36)		0.85(0.82–0.87)	0.29(0.28–0.29)
Mozambique	392(275–516)	6.28(4.4–8.26)		2506(1825–3184)	15.86(11.55–20.15)		8052(7318–8786)	28.25(25.68–30.83)		2.94(2.93–2.96)	3.24(3.2–3.27)
Namibia	71(52–90)	10.24(7.57–13.05)		307(199–414)	18.72(12.14–25.22)		633(575–692)	24.74(22.45–27.03)		1.91(1.88–1.94)	1.49(1.47–1.52)
Niger	171(127–225)	4.42(3.27–5.8)		1307(1049–1575)	11.31(9.08–13.63)		3654(3198–4110)	16.2(14.18–18.22)		2.97(2.93–3.01)	1.93(1.91–1.96)
Nigeria	4385(3742–5032)	9.35(7.98–10.73)		23006(19074–26846)	20.13(16.69–23.49)		43922(38328–49516)	23.69(20.67–26.71)		2.42(2.38–2.47)	0.89(0.86–0.92)
Rwanda	197(140–259)	5.99(4.26–7.86)		1143(1001–1285)	15.28(13.38–17.17)		3448(3117–3779)	28.66(25.91–31.41)		2.97(2.92–3.01)	3.62(3.58–3.65)
Sao Tome and Principe	6(4–8)	9.97(6.68–13.44)		31(26–36)	25.37(21.22–29.4)		69(61–78)	36.24(31.75–40.74)		2.96(2.94–2.98)	2(1.97–2.03)
Senegal	273(224–325)	7.59(6.22–9.03)		1682(1308–2049)	17.64(13.72–21.49)		4254(3781–4728)	27.09(24.08–30.11)		2.67(2.63–2.7)	2.41(2.36–2.45)
Seychelles	10(9–12)	23.01(20.06–25.91)		31(23–38)	32.3(24.62–39.58)		39(35–43)	34.75(31.46–38.03)		1.07(1.05–1.09)	0.4(0.39–0.41)
Sierra Leone	139(89–196)	6.53(4.19–9.2)		687(562–809)	15.28(12.49–17.99)		1607(1432–1782)	22.59(20.14–25.05)		2.69(2.66–2.71)	2.11(2.09–2.13)
South Africa	4922(4122–5694)	21.45(17.96–24.81)		10318(8627–11928)	24.1(20.15–27.86)		13722(12551–14894)	25.63(23.44–27.82)		0.36(0.34–0.38)	0.22(0.21–0.24)
South Sudan	166(84–257)	7.34(3.72–11.38)		840(466–1195)	14.82(8.23–21.08)		2127(1943–2311)	21.68(19.8–23.55)		2.22(2.2–2.24)	2.11(2.07–2.15)
Togo	124(87–169)	6.47(4.54–8.77)		901(769–1031)	18.57(15.85–21.26)		2140(1916–2363)	27.67(24.78–30.56)		3.35(3.32–3.38)	2.14(2.12–2.15)
Uganda	433(319–553)	5.49(4.04–7.01)		3408(2925–3883)	14.87(12.76–16.94)		12401(11049–13752)	29.85(26.6–33.1)		3.17(3.15–3.19)	4.01(3.96–4.06)
United Republic of Tanzania	1032(792–1306)	8.46(6.49–10.7)		6116(4688–7527)	18.8(14.41–23.14)		16016(14592–17439)	27.64(25.18–30.09)		2.53(2.5–2.55)	2.14(2.12–2.16)
Zambia	264(204–329)	7.63(5.9–9.51)		1800(1423–2167)	17.64(13.95–21.24)		6325(5702–6947)	34.34(30.96–37.72)		2.66(2.64–2.67)	3.67(3.63–3.71)
Zimbabwe	654(544–767)	14.12(11.73–16.56)		1572(1205–1928)	18.99(14.56–23.29)		2693(2470–2915)	20.51(18.82–22.21)		0.94(0.92–0.96)	0.45(0.42–0.47)
**Americas**											

Antigua and Barbuda	10(5–13)	23.57(13.28–32.92)		21(11–31)	29.93(15.53–43.49)		24(22–26)	30.66(27.92–33.39)		0.75(0.73–0.77)	0.13(0.12–0.15)
Argentina	6477(5650–7247)	30.79(26.86–34.45)		10996(9769–12212)	33.35(29.63–37.04)		14350(13138–15562)	37.18(34.04–40.32)		0.25(0.23–0.27)	0.51(0.48–0.53)
Bahamas	46(30–61)	25.46(16.82–33.77)		88(72–103)	28.73(23.5–33.64)		96(89–103)	27.96(25.83–30.1)		0.38(0.36–0.4)	-0.25(-0.26–-0.24)
Barbados	57(50–64)	31.33(27.46–35.13)		66(48–83)	29.86(21.59–37.51)		59(55–63)	26.38(24.5–28.27)		-0.15(-0.16–-0.13)	-0.68(-0.69–-0.67)
Belize	23(16–29)	25.44(18.09–32.45)		81(65–96)	30.07(24.23–35.64)		111(101–121)	30.4(27.67–33.13)		0.52(0.49–0.54)	0.03(0.01–0.04)
Bermuda	11(6–15)	25.03(14.07–34.99)		16(9–24)	30.74(16.45–44.31)		16(15–17)	30.62(27.99–33.25)		0.65(0.63–0.67)	-0.01(-0.03–0)
Bolivia (Plurinational State of)	945(743–1132)	24.89(19.57–29.81)		2802(2438–3152)	36.43(31.7–40.99)		5594(4916–6271)	53.76(47.24–60.27)		1.2(1.18–1.21)	1.98(1.95–2.01)
Brazil	21616(19884–23295)	24.84(22.85–26.77)		56190(50587–61745)	35.4(31.87–38.9)		74547(69177–79917)	42.09(39.06–45.12)		1.12(1.09–1.15)	0.88(0.87–0.89)
Canada	7057(6667–7448)	33.78(31.91–35.65)		11121(9913–12326)	35.26(31.43–39.08)		13063(12200–13926)	35.62(33.26–37.97)		0.14(0.11–0.16)	0.02(0.02–0.02)
Chile	2694(2378–3002)	31.04(27.39–34.58)		6196(5393–6961)	40.32(35.1–45.3)		8033(7290–8776)	45.95(41.7–50.2)		0.82(0.8–0.84)	0.59(0.57–0.6)
Colombia	4530(3908–5153)	24.15(20.83–27.47)		13997(12523–15426)	36.36(32.53–40.07)		21355(19330–23380)	45.61(41.28–49.93)		1.28(1.25–1.3)	1.14(1.12–1.15)
Costa Rica	533(421–643)	29.19(23.06–35.23)		1386(1156–1611)	35.93(29.96–41.75)		1682(1549–1815)	37.46(34.49–40.43)		0.65(0.63–0.68)	0.13(0.12–0.14)
Cuba	1504(1301–1699)	19.83(17.16–22.4)		3111(2704–3492)	34.65(30.12–38.89)		3854(3553–4155)	44.81(41.31–48.31)		1.77(1.75–1.8)	1.39(1.37–1.41)
Dominica	9(6–12)	21.79(15.31–28.04)		14(10–19)	27.85(18.69–36.86)		15(14–17)	29.48(26.67–32.29)		0.77(0.75–0.8)	0.25(0.23–0.27)
Dominican Republic	812(716–907)	21(18.52–23.46)		2567(2168–2948)	33.8(28.54–38.81)		4016(3653–4378)	42.99(39.11–46.86)		1.5(1.48–1.52)	1.2(1.19–1.22)
Ecuador	1781(1425–2111)	31.14(24.93–36.92)		4836(4378–5300)	39.21(35.5–42.97)		7365(6673–8057)	46.7(42.31–51.08)		0.72(0.71–0.74)	0.84(0.82–0.86)
El Salvador	823(652–998)	29.62(23.46–35.9)		1504(1228–1762)	34.71(28.32–40.65)		1893(1736–2051)	37.39(34.28–40.5)		0.49(0.48–0.51)	0.31(0.3–0.31)
Grenada	14(10–18)	23.28(16.35–30.27)		26(19–34)	29.86(21.35–38.02)		30(27–32)	31.04(28.21–33.87)		0.78(0.76–0.8)	0.2(0.19–0.22)
Guatemala	1079(913–1244)	24.91(21.07–28.71)		3871(3296–4386)	35.6(30.31–40.34)		7221(6599–7844)	45.25(41.35–49.15)		1.13(1.11–1.14)	1.19(1.18–1.19)
Guyana	89(65–113)	21.06(15.35–26.86)		148(115–179)	27.5(21.38–33.35)		187(172–203)	29.11(26.72–31.5)		0.83(0.81–0.86)	0.27(0.26–0.29)
Haiti	421(298–554)	11.9(8.42–15.65)		1327(987–1671)	18.6(13.83–23.42)		2252(2064–2439)	23.71(21.74–25.69)		1.41(1.39–1.42)	1.27(1.26–1.28)
Honduras	530(391–660)	22.62(16.69–28.14)		2228(1556–2819)	34.06(23.79–43.1)		4156(3745–4567)	44.19(39.82–48.56)		1.28(1.26–1.31)	1.23(1.22–1.24)
Jamaica	281(230–329)	20.47(16.75–23.99)		588(469–703)	27.42(21.88–32.76)		673(609–738)	30.57(27.63–33.5)		0.92(0.9–0.95)	0.51(0.5–0.51)
Mexico	14061(12788–15254)	31.59(28.73–34.27)		33383(31408–35241)	37.36(35.15–39.44)		42623(39121–46126)	38.53(35.37–41.7)		0.53(0.5–0.55)	0.06(0.05–0.07)
Nicaragua	507(394–619)	26.14(20.3–31.88)		1448(1035–1829)	33.31(23.82–42.09)		2204(2016–2393)	37.12(33.95–40.3)		0.76(0.74–0.78)	0.46(0.45–0.46)
Panama	371(297–441)	26.36(21.12–31.32)		1087(945–1218)	35.6(30.95–39.9)		1675(1531–1819)	41.8(38.21–45.39)		0.94(0.92–0.97)	0.74(0.72–0.75)

Paraguay	575(427–718)	27.2(20.23–33.97)		1596(1219–1954)	35.82(27.36–43.87)		2464(2256–2673)	42.53(38.93–46.12)		0.86(0.85–0.88)	0.81(0.79–0.82)
Peru	3187(2736–3622)	26.91(23.1–30.58)		9564(9111–10007)	40.79(38.86–42.68)		17763(15768–19758)	60.01(53.27–66.74)		1.31(1.29–1.33)	1.96(1.94–1.99)
Puerto Rico	719(550–883)	30.21(23.1–37.13)		923(677–1155)	33.84(24.81–42.35)		819(763–875)	32.19(29.98–34.39)		0.35(0.34–0.37)	-0.26(-0.27–-0.25)
Saint Kitts and Nevis	6(4–8)	26.5(17.61–35.02)		11(7–14)	29.86(19.45–39.87)		11(10–12)	28.94(26.02–31.87)		0.37(0.35–0.39)	-0.18(-0.19–-0.17)
Saint Lucia	17(15–20)	23.36(19.44–27.21)		41(35–47)	29.53(24.94–33.84)		42(39–46)	28.72(26.26–31.18)		0.74(0.72–0.76)	-0.15(-0.16–-0.15)
Saint Vincent and the Grenadines	14(9–18)	22.15(14.76–29.12)		22(17–26)	28.72(21.92–35.18)		22(20–24)	29.6(26.93–32.27)		0.81(0.79–0.83)	0.15(0.14–0.17)
Suriname	50(32–67)	21.47(13.8–28.75)		124(84–160)	29.02(19.76–37.53)		161(148–174)	30.85(28.33–33.38)		0.95(0.93–0.96)	0.32(0.31–0.34)
Trinidad and Tobago	205(163–244)	26.91(21.49–32.02)		355(275–435)	30.44(23.61–37.29)		357(331–384)	29.35(27.2–31.5)		0.38(0.36–0.4)	-0.25(-0.26–-0.25)
United States of America	61639(58789–64413)	32.66(31.15–34.13)		83415(74340–92330)	31.25(27.85–34.59)		93379(87218–99540)	31.19(29.13–33.25)		-0.13(-0.15–-0.11)	-0.07(-0.08–-0.06)
Uruguay	605(471–733)	28.13(21.91–34.07)		902(719–1084)	34.65(27.61–41.64)		1101(1013–1189)	39.89(36.7–43.08)		0.66(0.64–0.67)	0.73(0.71–0.75)
Venezuela (Bolivarian Republic of)	3456(2756–4099)	31.4(25.04–37.25)		5882(5050–6705)	30.95(26.57–35.28)		5632(5239–6024)	24.74(23.02–26.47)		-0.05(-0.08–-0.03)	-1.25(-1.26–-1.25)
**Europe**											
Albania	731(617–839)	36.26(30.63–41.61)		898(793–999)	40.13(35.44–44.66)		846(791–901)	40.43(37.81–43.05)		0.32(0.31–0.33)	0.06(0.05–0.08)
Andorra	13(11–15)	32.06(26.09–37.74)		22(15–28)	32.73(22.64–41.72)		24(22–26)	32.27(29.65–34.89)		0.07(0.05–0.09)	-0.06(-0.08–-0.04)
Armenia	592(474–703)	25.71(20.62–30.57)		704(590–816)	31.96(26.79–37.01)		738(680–795)	33.92(31.28–36.57)		0.68(0.67–0.7)	0.3(0.26–0.35)
Austria	1912(1753–2072)	31.65(29.01–34.3)		2421(2026–2795)	32.27(27.01–37.26)		2513(2341–2685)	33.01(30.75–35.26)		0.06(0.04–0.07)	0.12(0.11–0.13)
Azerbaijan	1192(985–1388)	27.16(22.45–31.63)		2719(2348–3071)	36.24(31.29–40.93)		3267(2995–3539)	36.92(33.84–39.99)		0.9(0.89–0.92)	0.1(0.08–0.12)
Belarus	2425(1788–3010)	32.78(24.17–40.69)		2694(2405–2969)	36.69(32.76–40.44)		2909(2726–3091)	41.74(39.12–44.36)		0.35(0.35–0.36)	0.7(0.69–0.72)
Belgium	2791(2648–2931)	35.9(34.06–37.7)		3250(2800–3672)	34.87(30.05–39.4)		3251(3046–3457)	32.6(30.54–34.66)		-0.09(-0.1–-0.08)	-0.38(-0.4–-0.36)
Bosnia and Herzegovina	1095(904–1279)	34.96(28.86–40.81)		1005(780–1221)	37.55(29.15–45.63)		897(836–959)	38.21(35.61–40.82)		0.24(0.19–0.3)	0.08(0.06–0.1)
Bulgaria	2274(1904–2640)	34.26(28.68–39.77)		1951(1726–2162)	34.62(30.63–38.37)		1735(1607–1863)	35.23(32.62–37.83)		0.03(0.02–0.04)	0.14(0.11–0.17)
Croatia	1330(1174–1479)	36.16(31.94–40.21)		1122(977–1268)	34.77(30.28–39.28)		1200(1079–1322)	40.62(36.49–44.74)		-0.12(-0.15–-0.09)	0.9(0.83–0.96)
Cyprus	187(155–216)	33.55(27.92–38.82)		351(235–457)	32.52(21.78–42.38)		387(361–412)	31.2(29.15–33.25)		-0.1(-0.1–-0.09)	-0.23(-0.24–-0.22)
Czechia	2829(2661–2988)	37.44(35.22–39.55)		3167(2804–3495)	36.63(32.44–40.43)		3214(3028–3401)	37.58(35.4–39.77)		-0.07(-0.08–-0.05)	0.18(0.15–0.2)
Denmark	1155(1082–1225)	28.55(26.74–30.29)		1611(1425–1790)	33.93(30–37.7)		1764(1633–1895)	35.06(32.45–37.66)		0.54(0.52–0.56)	0.17(0.16–0.18)
Estonia	365(321–407)	31.61(27.81–35.18)		403(336–467)	37.08(30.89–42.92)		493(456–531)	46.63(43.08–50.19)		0.5(0.49–0.51)	1.27(1.23–1.3)

Finland	1353(1280–1426)	35.21(33.32–37.11)		1677(1480–1867)	36.96(32.63–41.16)		1725(1608–1842)	37.07(34.55–39.58)		0.15(0.13–0.17)	0.02(0.01–0.03)
France	13930(12726–15117)	32.4(29.6–35.16)		14575(13162–15930)	27.75(25.06–30.33)		12850(12011–13689)	23.03(21.52–24.53)		-0.48(-0.49–-0.48)	-0.97(-0.98–-0.96)
Georgia	1012(737–1280)	26.56(19.34–33.59)		909(743–1076)	31.8(25.98–37.62)		965(897–1032)	32.14(29.89–34.39)		0.56(0.55–0.57)	0.04(0–0.08)
Germany	23005(22039–23933)	35.71(34.21–37.15)		25487(22024–28958)	36.35(31.41–41.3)		24870(23148–26592)	36.46(33.94–38.99)		0.06(0.05–0.06)	0.01(0–0.02)
Greece	2803(2403–3192)	35.9(30.78–40.88)		3050(2619–3448)	35.25(30.27–39.85)		2852(2639–3064)	35.52(32.87–38.16)		-0.06(-0.07–-0.05)	0.06(0.04–0.09)
Greenland	10(8–12)	25.44(19.28–31.31)		14(11–16)	32.51(27.04–37.79)		15(14–17)	36.58(33.12–40.04)		0.77(0.75–0.78)	0.65(0.64–0.67)
Hungary	2881(2667–3086)	37.02(34.28–39.66)		2528(2182–2877)	31.7(27.36–36.07)		2350(2191–2509)	30.99(28.89–33.09)		-0.48(-0.51–-0.46)	-0.15(-0.17–-0.12)
Iceland	57(53–61)	31.95(29.5–34.38)		110(87–131)	37.09(29.3–44.23)		136(126–146)	38.72(35.9–41.55)		0.47(0.45–0.49)	0.23(0.22–0.25)
Ireland	874(769–975)	37.07(32.63–41.33)		1470(1235–1689)	37.58(31.57–43.16)		1728(1620–1836)	36.34(34.06–38.62)		0.05(0.03–0.07)	-0.23(-0.26–-0.2)
Israel	972(894–1047)	33.97(31.25–36.59)		1999(1707–2278)	32.58(27.82–37.12)		2706(2519–2893)	32.67(30.42–34.93)		-0.13(-0.14–-0.12)	-0.01(-0.03–0.01)
Italy	15849(15164–16516)	35.16(33.64–36.64)		18031(16077–19935)	35.7(31.83–39.47)		16422(15347–17497)	34.03(31.8–36.26)		0.05(0.04–0.06)	-0.21(-0.22–-0.2)
Kazakhstan	2822(2349–3264)	26.19(21.8–30.29)		4721(4133–5269)	35.77(31.31–39.92)		7440(6794–8086)	43.54(39.76–47.32)		0.98(0.97–0.99)	1.02(0.98–1.06)
Kyrgyzstan	603(522–683)	23.89(20.68–27.07)		1351(1081–1612)	31.28(25.03–37.33)		1777(1639–1914)	29.39(27.12–31.67)		0.85(0.84–0.86)	-0.33(-0.34–-0.31)
Latvia	622(515–725)	31.44(26.03–36.64)		569(440–692)	37.36(28.89–45.48)		632(589–675)	44.91(41.86–47.95)		0.54(0.54–0.55)	1.04(1.02–1.07)
Lithuania	968(891–1045)	35.84(33–38.68)		834(622–1037)	36.05(26.87–44.79)		810(760–860)	37.88(35.55–40.21)		0.03(0.01–0.04)	0.32(0.28–0.36)
Luxembourg	99(79–117)	32.62(26.02–38.65)		186(154–217)	35.09(29.02–40.98)		231(216–247)	36.66(34.21–39.11)		0.23(0.21–0.24)	0.23(0.2–0.26)
Malta	99(84–112)	37.17(31.78–42.32)		151(120–181)	33.82(26.85–40.59)		148(139–158)	30.78(28.83–32.74)		-0.3(-0.3–-0.29)	-0.55(-0.57–-0.53)
Montenegro	126(96–153)	29.16(22.18–35.51)		162(115–205)	33.84(24.1–42.8)		168(156–179)	35.83(33.41–38.25)		0.47(0.45–0.48)	0.32(0.27–0.37)
Netherlands (Kingdom of the)	3776(3498–4052)	32.36(29.98–34.73)		5048(4183–5833)	34.67(28.73–40.06)		5179(4787–5571)	32.93(30.43–35.42)		0.22(0.2–0.24)	-0.27(-0.28–-0.26)
North Macedonia	499(384–605)	35.73(27.48–43.27)		519(340–679)	35.51(23.25–46.46)		499(465–533)	36.1(33.65–38.55)		-0.02(-0.04–0.01)	0.06(0.03–0.08)
Norway	1080(1010–1148)	33.19(31.04–35.28)		1744(1496–1976)	40.12(34.43–45.47)		2050(1915–2185)	42.11(39.33–44.88)		0.6(0.58–0.62)	0.25(0.24–0.26)
Poland	8880(8243–9509)	33.17(30.79–35.52)		11625(10210–12986)	37.07(32.56–41.41)		12955(11930–13980)	42.61(39.24–45.99)		0.35(0.33–0.37)	0.71(0.68–0.73)
Portugal	2820(2525–3104)	37.94(33.97–41.77)		3107(2568–3631)	35.5(29.35–41.49)		2563(2386–2740)	30.17(28.09–32.26)		-0.2(-0.22–-0.19)	-0.9(-0.91–-0.89)
Republic of Moldova	951(748–1144)	31.68(24.92–38.12)		909(820–993)	39.17(35.35–42.78)		1126(1041–1211)	53.75(49.68–57.82)		0.67(0.66–0.68)	1.72(1.7–1.75)
Romania	5327(4799–5843)	32.38(29.17–35.52)		5094(4299–5886)	32.98(27.83–38.11)		5313(4792–5835)	37.33(33.67–40.99)		0.07(0.03–0.11)	0.65(0.57–0.72)

Russian Federation	34656(32853–36470)	31.91(30.25–33.58)		42378(36510–47796)	36.76(31.67–41.46)		54519(50042–58995)	47.35(43.47–51.24)		0.45(0.43–0.47)	1.36(1.32–1.4)
Serbia	2103(1901–2296)	34.85(31.5–38.06)		1906(1576–2235)	33.96(28.09–39.83)		1691(1561–1821)	33.64(31.05–36.22)		-0.08(-0.08–-0.07)	-0.04(-0.06–-0.02)
Slovakia	1303(1177–1419)	35.55(32.11–38.71)		1591(1287–1880)	35.86(29.02–42.39)		1643(1528–1758)	37.52(34.89–40.15)		0.03(0–0.05)	0.2(0.17–0.23)
Slovenia	551(508–594)	36.83(33.99–39.72)		671(607–733)	38.65(34.99–42.23)		631(584–678)	36.35(33.66–39.04)		0.15(0.14–0.17)	-0.33(-0.35–-0.31)
Spain	10818(10272–11358)	36.85(34.99–38.69)		14973(13173–16717)	37.6(33.08–41.98)		14822(13762–15883)	36.61(33.99–39.23)		0.06(0.05–0.08)	-0.12(-0.14–-0.11)
Sweden	2156(2041–2270)	32.24(30.52–33.94)		3138(2691–3570)	37.85(32.46–43.06)		3768(3507–4029)	40.83(38.01–43.66)		0.5(0.49–0.51)	0.4(0.38–0.42)
Switzerland	1698(1539–1846)	31.91(28.92–34.7)		2238(1957–2511)	31.03(27.13–34.82)		2350(2195–2505)	30.09(28.1–32.07)		-0.09(-0.09–-0.08)	-0.2(-0.21–-0.18)
Tajikistan	582(426–728)	21.5(15.75–26.9)		2036(1735–2314)	34.35(29.27–39.04)		3919(3619–4219)	43.98(40.61–47.34)		1.48(1.46–1.49)	1.33(1.3–1.35)
Turkey	10381(9592–11183)	32.11(29.67–34.59)		22127(19607–24635)	34.59(30.65–38.51)		25735(23582–27887)	35.03(32.1–37.97)		0.24(0.23–0.25)	0.09(0.06–0.11)
Turkmenistan	459(351–564)	23.2(17.76–28.49)		1643(1447–1832)	35.45(31.22–39.53)		2645(2458–2833)	41.45(38.52–44.39)		1.33(1.32–1.34)	0.82(0.81–0.83)
Ukraine	11939(9092–14682)	30.86(23.5–37.95)		11564(9841–13240)	34.29(29.18–39.26)		12620(11751–13489)	40.73(37.92–43.53)		0.32(0.29–0.35)	1.01(0.98–1.04)
United Kingdom	15439(14932–15951)	35(33.85–36.16)		19498(17975–20977)	36.25(33.42–39)		21708(20203–23214)	35.84(33.36–38.33)		0.11(0.1–0.12)	-0.1(-0.12–-0.08)
Uzbekistan	2331(1906–2754)	21.16(17.3–25)		7459(6523–8332)	32.89(28.76–36.74)		12370(11390–13351)	40.64(37.42–43.86)		1.39(1.37–1.4)	1.11(1.05–1.17)
**Eastern Mediterranean**											
Afghanistan	402(225–611)	7.32(4.09–11.12)		5571(4656–6470)	27.77(23.21–32.25)		23213(20385–26042)	64.28(56.45–72.12)		4.25(4.22–4.27)	4.52(4.49–4.55)
Bahrain	92(76–108)	28.93(23.72–33.87)		400(278–512)	33.64(23.37–43.04)		561(509–613)	35.96(32.61–39.31)		0.48(0.46–0.5)	0.36(0.34–0.37)
Djibouti	24(10–39)	8.51(3.62–13.75)		131(51–206)	18.01(7–28.44)		255(234–276)	25.93(23.8–28.06)		2.37(2.35–2.39)	2.05(2.03–2.07)
Egypt	9920(8635–11174)	32.11(27.95–36.17)		20558(17409–23561)	29.64(25.1–33.97)		25763(23417–28110)	25.84(23.49–28.19)		-0.25(-0.27–-0.23)	-0.74(-0.76–-0.72)
Iran (Islamic Republic of)	7747(6738–8733)	26.4(22.96–29.76)		24070(22437–25637)	37.01(34.5–39.42)		37881(34971–40792)	47.03(43.42–50.64)		1.06(1.05–1.07)	1.22(1.21–1.23)
Iraq	2348(1775–2890)	28.01(21.17–34.48)		8047(6472–9492)	32.56(26.19–38.41)		14508(13390–15626)	36.21(33.42–39)		0.47(0.47–0.48)	0.56(0.54–0.57)
Jordan	579(501–656)	33.3(28.81–37.75)		2187(1917–2440)	31.19(27.35–34.8)		2774(2582–2966)	27.15(25.27–29.03)		-0.2(-0.23–-0.18)	-0.78(-0.79–-0.77)
Kuwait	351(308–392)	35.72(31.4–39.91)		1310(1099–1513)	36.92(30.98–42.63)		1896(1715–2077)	40.91(37–44.81)		0.11(0.08–0.14)	0.49(0.47–0.51)
Lebanon	659(564–752)	32.1(27.49–36.67)		1362(1142–1566)	35.18(29.49–40.46)		1832(1692–1972)	37.16(34.31–40)		0.28(0.26–0.3)	0.32(0.3–0.33)
Libya	648(499–793)	28.57(22.01–34.98)		1473(1082–1851)	30.93(22.72–38.87)		2001(1861–2142)	30.83(28.66–33)		0.25(0.21–0.29)	-0.05(-0.06–-0.03)
Morocco	3099(2636–3539)	24(20.42–27.41)		8547(7257–9763)	33.4(28.36–38.15)		11773(10984–12563)	37.07(34.59–39.56)		1.03(1.02–1.05)	0.57(0.56–0.59)

State of Palestine	305(260–347)	32.33(27.56–36.76)		895(792–997)	30.67(27.12–34.16)		1326(1231–1422)	28.29(26.25–30.32)		-0.17(-0.18–-0.15)	-0.49(-0.5–-0.48)
Oman	242(213–270)	27.09(23.87–30.22)		1222(1025–1402)	36.75(30.83–42.15)		2396(2155–2636)	45.88(41.28–50.48)		0.95(0.92–0.99)	1.15(1.12–1.18)
Pakistan	6098(5069–7163)	10.54(8.76–12.38)		42269(36861–47470)	30.8(26.86–34.59)		122132(109335–134930)	59.9(53.62–66.18)		3.4(3.37–3.44)	3.57(3.53–3.6)
Qatar	109(85–131)	36.19(28.19–43.52)		834(617–1033)	34.9(25.84–43.25)		973(868–1078)	32.31(28.83–35.8)		-0.11(-0.15–-0.07)	-0.44(-0.47–-0.42)
Saudi Arabia	1935(1832–2036)	35.19(33.33–37.04)		7305(5717–8751)	31.93(24.99–38.25)		9127(8295–9959)	28.79(26.16–31.41)		-0.3(-0.33–-0.26)	-0.55(-0.57–-0.53)
Somalia	307(152–470)	9.24(4.58–14.15)		1523(889–2132)	18.44(10.77–25.82)		3976(3641–4311)	25.67(23.51–27.83)		2.18(2.16–2.2)	1.88(1.84–1.92)
Sudan	1382(928–1865)	13.25(8.9–17.88)		5185(3922–6372)	20.04(15.16–24.63)		9047(8231–9864)	21.55(19.6–23.49)		1.3(1.27–1.33)	0.52(0.5–0.54)
Syrian Arab Republic	1982(1641–2310)	33.93(28.1–39.55)		4961(3629–6248)	36.32(26.57–45.74)		8844(8155–9533)	38.22(35.25–41.2)		0.2(0.19–0.22)	0.29(0.26–0.31)
Tunisia	1175(994–1353)	25.24(21.34–29.06)		2674(1934–3369)	31.06(22.47–39.14)		3459(3180–3738)	33.53(30.83–36.24)		0.66(0.64–0.67)	0.42(0.4–0.44)
United Arab Emirates	447(375–516)	35.87(30.09–41.39)		3392(2902–3843)	40.9(34.99–46.33)		4767(4277–5258)	43.6(39.11–48.08)		0.42(0.4–0.45)	0.46(0.45–0.47)
Yemen	797(611–984)	13.52(10.36–16.69)		4443(3142–5679)	22.24(15.73–28.43)		11299(10379–12219)	32.65(29.99–35.3)		1.57(1.55–1.59)	2.03(2.02–2.05)
**South-East Asia**											
Bangladesh	1622(1146–2183)	2.86(2.02–3.85)		24116(21690–26530)	21.87(19.67–24.06)		113327(97651–129003)	77.35(66.65–88.05)		6.57(6.53–6.62)	6.93(6.89–6.98)
Bhutan	32(20–44)	10.67(6.79–14.84)		194(173–215)	34.27(30.54–37.87)		446(403–489)	64.45(58.25–70.66)		3.7(3.68–3.73)	3.48(3.47–3.49)
Democratic People's Republic of Korea	1406(667–2192)	9.87(4.68–15.39)		5508(3068–7765)	27.09(15.09–38.19)		9508(8780–10237)	44.69(41.27–48.12)		3.21(3.19–3.23)	2.78(2.77–2.79)
India	23634(21152–26213)	4.95(4.43–5.49)		218007(200275–235838)	22.13(20.33–23.94)		689885(615524–764246)	55.99(49.96–62.03)		4.78(4.74–4.82)	5.1(5.07–5.14)
Indonesia	8831(7426–10236)	8.36(7.03–9.69)		53279(46590–59657)	27.4(23.96–30.68)		135121(121319–148923)	57.54(51.66–63.41)		3.79(3.76–3.82)	4.07(4.04–4.1)
Maldives	13(9–16)	12.16(8.78–15.62)		142(128–156)	35.65(31.99–39.14)		287(257–317)	60.39(54.09–66.68)		3.42(3.39–3.44)	2.94(2.91–2.97)
Myanmar	1942(1291–2647)	8.5(5.65–11.59)		7546(6015–9066)	19.96(15.91–23.98)		13043(11903–14182)	29.87(27.26–32.48)		2.71(2.69–2.73)	2.25(2.25–2.26)
Nepal	528(360–732)	5.18(3.53–7.18)		4439(3938–4919)	23.02(20.42–25.51)		16550(13833–19266)	69(57.67–80.32)		4.78(4.76–4.79)	6.01(5.97–6.05)
Sri Lanka	755(534–985)	7.54(5.34–9.84)		4719(4189–5228)	28.49(25.29–31.56)		9496(8694–10298)	49.88(45.67–54.1)		4.24(4.2–4.27)	3.11(3.09–3.14)
Thailand	4829(4255–5396)	13.88(12.23–15.51)		18055(16046–19953)	31.1(27.64–34.37)		27416(24967–29865)	46.04(41.93–50.15)		2.56(2.54–2.58)	2.13(2.11–2.15)
Timor-Leste	12(7–18)	3.01(1.79–4.58)		87(68–106)	10.89(8.49–13.36)		342(308–376)	28.91(26.02–31.79)		4.1(4.08–4.13)	5.54(5.53–5.55)
**Western Pacific**											

American Samoa	7(6–8)	26.16(23.18–29.07)		5(3–7)	16.16(8.65–23.49)		4(4–5)	14.04(12.55–15.53)		-1.5(-1.51–-1.48)	-0.83(-0.83–-0.82)
Australia	4199(3996–4395)	33.49(31.87–35.05)		7112(6288–7913)	34.72(30.7–38.63)		8387(7805–8970)	34.19(31.81–36.56)		0.11(0.1–0.12)	-0.14(-0.15–-0.13)
Brunei Darussalam	27(18–37)	17.77(11.44–24.28)		131(110–150)	38.54(32.46–44.31)		208(189–227)	51.6(46.96–56.25)		2.44(2.42–2.47)	1.6(1.59–1.61)
Cambodia	166(114–228)	4.47(3.06–6.13)		2279(2039–2522)	20.75(18.56–22.96)		7165(6553–7777)	49.56(45.32–53.79)		4.94(4.9–4.97)	4.9(4.89–4.92)
China	80755(76627–84733)	10.76(10.21–11.29)		347527(321369–373346)	30.69(28.38–32.97)		617766(539386–696146)	51.89(45.31–58.47)		3.34(3.3–3.37)	2.85(2.8–2.9)
Cook Islands	3(2–4)	32.25(24.19–39.68)		2(1–3)	20.01(13.43–26.7)		1(1–1)	16.04(13.99–18.1)		-1.48(-1.49–-1.48)	-1.25(-1.25–-1.25)
Fiji	126(108–144)	29.37(25.19–33.48)		197(144–247)	32.04(23.43–40.1)		230(214–246)	32.37(30.14–34.61)		0.27(0.26–0.28)	0.05(0.04–0.06)
French Polynesia	39(28–50)	33.82(24.05–42.64)		54(35–73)	25.72(16.42–34.67)		51(47–54)	21.17(19.71–22.64)		-0.85(-0.86–-0.84)	-1.07(-1.07–-1.07)
Japan	17150(16561–17729)	18.06(17.44–18.67)		22600(20335–24821)	21.16(19.04–23.24)		22223(20866–23580)	22.65(21.27–24.04)		0.5(0.49–0.51)	0.41(0.4–0.42)
Kiribati	14(12–16)	35.33(30.03–40.48)		25(20–30)	32.8(26.12–39.21)		34(30–37)	31.22(28.37–34.07)		-0.23(-0.24–-0.23)	-0.27(-0.27–-0.26)
Lao People's Democratic Republic	160(104–222)	7.5(4.89–10.38)		1047(792–1284)	21.89(16.56–26.86)		2721(2510–2933)	41.87(38.62–45.13)		3.41(3.39–3.42)	3.6(3.58–3.61)
Malaysia	1746(1449–2038)	17.3(14.36–20.19)		8276(7285–9217)	32.99(29.04–36.74)		13776(12627–14925)	41.71(38.23–45.19)		2.03(2–2.06)	1.25(1.24–1.27)
Marshall Islands	7(5–8)	34.07(27.13–40.89)		6(5–8)	27.14(21.89–32.57)		4(3–4)	21.56(19.08–24.04)		-0.71(-0.71–-0.7)	-1.22(-1.23–-1.21)
Micronesia (Federated States of)	15(11–18)	31.12(23.41–38.5)		17(11–22)	24.31(16.56–32.07)		16(14–17)	19.43(17.68–21.18)		-0.76(-0.77–-0.75)	-1.22(-1.23–-1.21)
Mongolia	259(210–305)	23.05(18.73–27.19)		732(643–818)	34.45(30.26–38.49)		1225(1127–1323)	41.25(37.95–44.55)		1.27(1.26–1.28)	0.92(0.87–0.97)
Nauru	1(1–1)	23.11(20.53–25.61)		1(1–2)	18.57(12.75–24.37)		1(1–2)	16.31(13.45–19.17)		-0.68(-0.7–-0.66)	-0.68(-0.69–-0.66)
New Zealand	811(753–867)	33.31(30.95–35.61)		1388(1283–1488)	34.85(32.21–37.35)		1617(1503–1730)	35.45(32.97–37.94)		0.14(0.12–0.16)	0.03(0.02–0.04)
Niue	0(0–1)	31.19(21.84–39.94)		0(0–0)	22.32(14.98–29.45)		0(0–0)	19(15.05–22.95)		-1.04(-1.07–-1.02)	-0.94(-0.97–-0.92)
Palau	3(3–4)	35.72(26.22–44.62)		4(3–5)	30.97(24.29–37.25)		4(3–4)	28.8(25.76–31.83)		-0.44(-0.45–-0.44)	-0.46(-0.46–-0.45)
Papua New Guinea	506(412–594)	25.28(20.61–29.7)		1944(1330–2505)	31.86(21.79–41.05)		3103(2889–3318)	35.01(32.59–37.42)		0.73(0.72–0.74)	0.48(0.48–0.49)
Philippines	3706(3281–4150)	11.42(10.11–12.79)		19096(16024–22057)	25.86(21.7–29.87)		38841(35052–42629)	40.1(36.18–44.01)		2.58(2.56–2.61)	2.44(2.43–2.45)

Republic of Korea	5920(5446–6388)	19.73(18.15–21.29)		13567(12806–14307)	30.47(28.76–32.13)		15861(14564–17158)	35.72(32.8–38.64)		1.37(1.34–1.4)	0.87(0.86–0.88)
Samoa	33(31–36)	38.53(35.67–41.33)		31(23–38)	25.82(19.32–32.13)		33(29–37)	21.76(19.31–24.2)		-1.24(-1.25–-1.23)	-0.84(-0.85–-0.83)
Singapore	486(449–522)	21.7(20.04–23.28)		1357(1131–1563)	28.48(23.74–32.8)		1737(1606–1869)	32.64(30.17–35.12)		0.87(0.84–0.89)	1(1–1.01)
Solomon Islands	40(29–50)	25.86(18.78–32.31)		151(123–177)	34.42(28.03–40.56)		313(286–340)	43.89(40.11–47.66)		0.9(0.89–0.9)	1.34(1.33–1.36)
Tokelau	0(0–0)	29.27(21.1–36.91)		0(0–0)	20.58(13.94–27.12)		0(0–1)	17.4(13.9–20.9)		-1.1(-1.12–-1.07)	-0.97(-0.99–-0.95)
Tonga	18(14–22)	34.89(27.31–42)		12(9–14)	19.06(14.34–23.8)		8(7–9)	11.71(10.35–13.07)		-1.87(-1.89–-1.85)	-2.6(-2.61–-2.58)
Tuvalu	2(1–2)	30.52(20.23–40.07)		1(1–2)	21.6(15.03–28.1)		1(1–1)	17.33(14.7–19.96)		-1.07(-1.08–-1.06)	-1.2(-1.21–-1.19)
Vanuatu	20(16–23)	26.79(21.94–31.44)		51(37–64)	29.39(21.37–36.73)		84(76–91)	30.04(27.46–32.62)		0.29(0.28–0.3)	0.07(0.07–0.08)
Viet Nam	881(759–1011)	2.45(2.11–2.81)		12044(10627–13497)	16.83(14.85–18.86)		43382(37798–48967)	50.8(44.27–57.34)		6.22(6.15–6.3)	6.19(6.16–6.22)

Table S2. The prevalence of obesity and average annual percentage change (AAPCs) during 1990–2040 by geographical regions and countries

Characteristics	1990		2022		2040		1990-2022	2023-2040
	Cases, NO, $\times 10^3$(95% CI)	Prevalence (%) No. (95% CI))	Cases, NO, $\times 10^3$(95% CI)	Prevalence (%) No. (95% CI)	Cases, NO, $\times 10^3$(95% CI)	Prevalence (%) No. (95% CI)	AAPC (%) No. (95% CI)	AAPC(%) No. (95% CI)
**Africa**								
Algeria	1113(837–1436)	8.59(6.46–11.08)	7124(6137–8140)	24.25(20.89–27.71)	17402(15974–18830)	42.82(39.31–46.34)	3.3(3.28–3.31)	3.05(3.02–3.08)
Angola	150(78–262)	2.64(1.38–4.61)	1831(1204–2583)	10.54(6.93–14.87)	8639(7598–9680)	27.08(23.81–30.34)	4.42(4.37–4.46)	5.15(5.1–5.2)
Benin	44(31–58)	1.74(1.25–2.33)	698(572–840)	9.89(8.1–11.89)	3254(2756–3751)	27.52(23.31–31.73)	5.58(5.51–5.66)	5.67(5.62–5.71)
Botswana	31(21–44)	4.98(3.29–7.07)	262(210–318)	17.46(14.03–21.21)	755(671–839)	35.64(31.67–39.62)	4(3.97–4.03)	4.34(4.34–4.34)
Burkina Faso	22(15–31)	0.51(0.36–0.72)	688(583–798)	6.1(5.17–7.08)	5438(4617–6259)	28.05(23.82–32.29)	8.06(7.98–8.15)	8.6(8.56–8.64)
Burundi	19(9–37)	0.75(0.36–1.46)	284(164–480)	4.53(2.62–7.66)	2105(1847–2363)	18.33(16.08–20.57)	5.77(5.73–5.81)	7.93(7.87–7.99)
Cabo Verde	5(3–8)	2.81(1.75–4.27)	53(45–61)	15.09(12.97–17.36)	150(132–168)	33.38(29.35–37.4)	5.39(5.35–5.43)	4.44(4.42–4.46)
Cameroon	147(108–195)	2.74(2.02–3.64)	1892(1588–2228)	13.35(11.2–15.72)	7308(6151–8466)	29.95(25.2–34.69)	5.07(5.04–5.1)	4.36(4.32–4.4)
Central African Republic	18(12–27)	1.25(0.81–1.87)	168(122–225)	7.67(5.56–10.25)	1140(1001–1280)	27.55(24.19–30.92)	5.82(5.75–5.9)	7.26(7.21–7.3)
Chad	22(13–36)	0.79(0.48–1.3)	482(318–709)	5.71(3.76–8.39)	2741(2319–3164)	17.15(14.5–19.79)	6.37(6.3–6.44)	6.15(6.12–6.18)
Comoros	6(4–8)	2.8(1.94–3.83)	73(57–90)	15.55(12.22–19.19)	315(278–352)	45.02(39.74–50.3)	5.5(5.44–5.56)	6.06(6.01–6.1)
Congo	27(21–34)	2.32(1.81–2.91)	251(182–331)	7.96(5.77–10.49)	770(667–873)	14.48(12.55–16.41)	3.92(3.87–3.97)	3.3(3.24–3.35)
Cote d'Ivoire	104(75–140)	1.88(1.36–2.53)	1672(1328–2096)	10.59(8.41–13.28)	7657(6595–8719)	28.62(24.65–32.58)	5.55(5.51–5.6)	5.29(5.19–5.38)
Democratic Republic of the Congo	196(127–292)	1.08(0.7–1.61)	2799(2041–3664)	5.76(4.2–7.54)	17490(15411–19569)	19.57(17.24–21.9)	5.38(5.33–5.43)	6.79(6.75–6.84)
Equatorial Guinea	13(8–19)	5.06(3.03–7.71)	175(128–231)	17.16(12.57–22.64)	619(543–695)	38.14(33.48–42.8)	3.88(3.85–3.92)	4.43(4.39–4.48)
Eritrea	6(4–8)	0.58(0.38–0.85)	76(49–111)	4.2(2.69–6.12)	518(457–578)	17.57(15.53–19.62)	6.39(6.33–6.44)	8.3(8.23–8.37)
Eswatini	32(23–43)	8.06(5.74–10.9)	199(170–229)	27.31(23.38–31.46)	614(554–673)	64.01(57.85–70.18)	3.9(3.85–3.96)	4.76(4.72–4.79)
Ethiopia	66(41–104)	0.29(0.18–0.46)	1612(1191–2094)	2.41(1.78–3.13)	13891(12164–15618)	12.25(10.73–13.78)	6.81(6.67–6.96)	9.27(9.23–9.3)
Gabon	26(18–36)	5.1(3.54–7.01)	280(233–336)	20(16.65–23.97)	985(879–1091)	45.34(40.48–50.21)	4.36(4.34–4.39)	4.51(4.47–4.56)
Gambia	7(5–9)	1.32(0.9–1.84)	181(149–219)	13.22(10.82–15.98)	989(817–1161)	42.9(35.45–50.35)	7.49(7.44–7.54)	6.67(6.61–6.73)
Ghana	182(141–232)	2.46(1.9–3.14)	2296(1968–2651)	12.11(10.38–13.98)	7190(6069–8312)	25.24(21.3–29.17)	5.09(5.03–5.15)	4.04(4.01–4.07)
Guinea	35(23–51)	1.07(0.7–1.57)	603(463–770)	8.29(6.36–10.59)	3640(3153–4126)	30.51(26.43–34.59)	6.61(6.56–6.66)	7.34(7.3–7.38)
Guinea-Bissau	8(4–12)	1.65(0.94–2.66)	114(77–162)	10.08(6.8–14.39)	484(422–547)	26.45(23.03–29.86)	5.82(5.76–5.88)	5.46(5.42–5.5)

Kenya	170(125–227)	1.7(1.25–2.27)		3289(2887–3709)	11.04(9.69–12.45)		14636(12991–16282)	31.57(28.02–35.12)		6.03(5.99–6.07)	5.86(5.84–5.88)
Lesotho	63(49–78)	6.86(5.39–8.53)		258(216–303)	19.29(16.14–22.67)		737(675–799)	40.19(36.8–43.59)		3.28(3.26–3.31)	4.14(4.11–4.17)
Liberia	22(15–32)	2(1.35–2.86)		457(408–510)	16.21(14.45–18.08)		3887(3184–4591)	83.85(68.68–99.02)		6.74(6.68–6.81)	9.23(9.16–9.31)
Madagascar	29(18–44)	0.51(0.31–0.78)		628(422–934)	3.85(2.59–5.73)		4919(4309–5529)	18.42(16.14–20.71)		6.55(6.47–6.63)	8.98(8.92–9.04)
Malawi	44(31–62)	0.96(0.68–1.34)		665(532–814)	6.37(5.1–7.8)		3873(3463–4282)	20.74(18.55–22.93)		6.1(6.07–6.14)	6.69(6.67–6.71)
Mali	56(38–80)	1.3(0.88–1.86)		1062(822–1361)	9.95(7.7–12.76)		6128(5141–7115)	31.21(26.18–36.24)		6.56(6.49–6.62)	6.33(6.28–6.37)
Mauritania	38(26–52)	4.09(2.83–5.61)		508(432–599)	20.84(17.72–24.58)		2259(1947–2570)	52.24(45.04–59.44)		5.23(5.18–5.28)	5.07(5.03–5.1)
Mauritius	55(48–61)	7.75(6.89–8.66)		199(165–234)	19.47(16.12–22.86)		355(313–397)	35.06(30.92–39.21)		2.91(2.87–2.95)	3.25(3.22–3.28)
Mozambique	89(62–125)	1.43(1–2)		1387(1070–1757)	8.78(6.77–11.12)		9836(8786–10887)	34.52(30.83–38.2)		5.85(5.81–5.89)	7.77(7.7–7.85)
Namibia	34(26–42)	4.86(3.81–6.08)		251(192–317)	15.29(11.72–19.33)		701(631–771)	27.38(24.63–30.12)		3.65(3.62–3.68)	3.3(3.26–3.33)
Niger	31(22–41)	0.79(0.56–1.07)		616(505–743)	5.33(4.37–6.43)		3814(3177–4450)	16.91(14.09–19.73)		6.17(6.09–6.25)	6.56(6.5–6.62)
Nigeria	985(802–1196)	2.1(1.71–2.55)		12389(10400–14594)	10.84(9.1–12.77)		43304(37025–49584)	23.36(19.97–26.74)		5.25(5.21–5.29)	4.28(4.22–4.34)
Rwanda	16(10–24)	0.48(0.3–0.73)		342(288–403)	4.57(3.85–5.39)		2562(2268–2855)	21.29(18.86–23.73)		7.32(7.22–7.41)	8.83(8.79–8.87)
Sao Tome and Principe	2(1–2)	2.74(1.82–3.93)		18(16–21)	15.04(12.74–17.42)		65(55–75)	33.96(28.87–39.05)		5.46(5.42–5.51)	4.63(4.58–4.67)
Senegal	68(54–83)	1.88(1.51–2.32)		828(648–1026)	8.68(6.8–10.76)		3178(2750–3607)	20.24(17.52–22.97)		4.88(4.83–4.94)	4.96(4.93–5)
Seychelles	5(5–6)	11.85(10.28–13.51)		29(24–34)	30.27(25.1–35.62)		55(49–62)	49.24(43.4–55.08)		2.97(2.94–3)	2.88(2.86–2.91)
Sierra Leone	34(22–50)	1.58(1.01–2.34)		291(235–355)	6.48(5.23–7.89)		857(730–983)	12.05(10.27–13.83)		4.5(4.42–4.59)	3.43(3.41–3.45)
South Africa	3227(2793–3690)	14.06(12.17–16.08)		12857(11731–14034)	30.03(27.4–32.78)		22379(20329–24428)	41.8(37.97–45.62)		2.41(2.37–2.44)	1.77(1.77–1.78)
South Sudan	32(16–59)	1.44(0.7–2.61)		452(288–657)	7.98(5.08–11.6)		2762(2467–3057)	28.15(25.14–31.16)		5.52(5.46–5.57)	7.14(7.06–7.23)
Togo	24(16–34)	1.24(0.84–1.77)		507(437–580)	10.45(9.01–11.97)		2674(2317–3032)	34.59(29.97–39.21)		6.9(6.85–6.94)	6.73(6.69–6.77)
Uganda	68(47–96)	0.86(0.59–1.22)		1577(1362–1825)	6.88(5.94–7.96)		22977(19579–26374)	55.3(47.13–63.48)		6.71(6.65–6.77)	12.14(12.01–12.27)
United Republic of Tanzania	207(157–267)	1.7(1.29–2.19)		3705(3009–4496)	11.39(9.25–13.82)		26105(23134–29075)	45.05(39.92–50.17)		6.14(6.1–6.18)	7.77(7.71–7.83)
Zambia	56(41–73)	1.61(1.19–2.12)		961(779–1162)	9.42(7.64–11.39)		7374(6470–8277)	40.04(35.13–44.95)		5.68(5.65–5.72)	8.12(8.07–8.17)
Zimbabwe	215(173–261)	4.64(3.74–5.64)		1029(829–1250)	12.43(10.02–15.1)		3727(3410–4044)	28.39(25.98–30.81)		3.15(3.09–3.21)	4.55(4.51–4.59)
**Americas**											
Antigua and Barbuda	5(3–7)	11.53(6.72–17.78)		24(17–32)	34.06(24.17–44.25)		42(38–46)	53.26(48.06–58.46)		3.45(3.41–3.49)	2.51(2.49–2.53)
Argentina	2686(2270–3149)	12.77(10.79–14.97)		11879(10959–12806)	36.03(33.24–38.84)		30278(27542–33014)	78.44(71.35–85.53)		3.29(3.28–3.31)	4.34(4.3–4.37)
Bahamas	32(24–42)	17.95(13.46–23.23)		146(134–158)	47.61(43.76–51.69)		239(217–260)	69.63(63.39–75.87)		3.09(3.06–3.12)	2.12(2.1–2.13)

Barbados	27(24–31)	14.99(12.97–17.13)		85(72–98)	38.23(32.51–44.3)		131(120–141)	58.36(53.78–62.93)		2.98(2.95–3)	2.37(2.36–2.38)
Belize	15(11–19)	16.22(12.52–20.64)		112(101–124)	41.91(37.52–46.33)		230(210–250)	62.92(57.4–68.44)		3.01(2.98–3.05)	2.27(2.26–2.29)
Bermuda	5(3–8)	12.42(7.38–19.03)		18(13–24)	34.01(24.07–44.27)		27(24–29)	50.63(45.92–55.34)		3.2(3.17–3.22)	2.28(2.27–2.3)
Bolivia (Plurinational State of)	291(221–378)	7.66(5.82–9.95)		2138(1892–2400)	27.8(24.6–31.2)		7417(6719–8116)	71.29(64.58–78)		4.11(4.1–4.12)	5.18(5.15–5.2)
Brazil	6735(5970–7545)	7.74(6.86–8.67)		45650(41682–49650)	28.76(26.26–31.28)		125927(116023–135830)	71.1(65.5–76.69)		4.19(4.18–4.21)	5.02(5–5.04)
Canada	2925(2695–3159)	14(12.9–15.12)		8617(7765–9478)	27.32(24.62–30.05)		12374(11292–13455)	33.74(30.79–36.69)		2.11(2.07–2.14)	1.13(1.12–1.15)
Chile	1082(920–1256)	12.46(10.6–14.47)		6073(5441–6700)	39.52(35.41–43.6)		15286(13541–17031)	87.44(77.46–97.41)		3.67(3.65–3.69)	4.33(4.3–4.37)
Colombia	1197(960–1461)	6.38(5.12–7.79)		9182(8208–10190)	23.85(21.32–26.47)		22321(20089–24554)	47.67(42.9–52.44)		4.21(4.18–4.23)	3.81(3.78–3.84)
Costa Rica	190(142–244)	10.41(7.79–13.38)		1234(1073–1402)	31.97(27.81–36.33)		2487(2281–2692)	55.37(50.8–59.95)		3.56(3.54–3.59)	2.98(2.97–2.99)
Cuba	506(425–595)	6.67(5.6–7.85)		2114(1858–2386)	23.54(20.69–26.57)		3577(3345–3809)	41.59(38.9–44.29)		4.02(3.99–4.05)	3.16(3.14–3.17)
Dominica	5(3–6)	10.71(7.78–14.22)		16(13–20)	31.5(25.31–37.89)		28(25–31)	53.26(47.66–58.86)		3.44(3.4–3.48)	2.97(2.95–2.98)
Dominican Republic	289(249–334)	7.47(6.44–8.63)		2212(1941–2494)	29.12(25.55–32.83)		5182(4718–5645)	55.47(50.51–60.43)		4.34(4.32–4.37)	3.5(3.49–3.51)
Ecuador	478(349–638)	8.36(6.1–11.15)		3328(2991–3654)	26.98(24.25–29.63)		9246(8572–9920)	58.62(54.35–62.9)		3.73(3.73–3.74)	4.32(4.29–4.34)
El Salvador	299(223–385)	10.74(8.02–13.84)		1296(1112–1491)	29.89(25.65–34.39)		2508(2297–2719)	49.52(45.35–53.69)		3.24(3.22–3.27)	2.77(2.76–2.77)
Grenada	6(4–9)	10.49(7.36–14.18)		27(22–32)	30.49(24.82–36.33)		49(44–53)	51.02(45.98–56.06)		3.39(3.37–3.41)	2.91(2.89–2.94)
Guatemala	292(231–360)	6.73(5.33–8.31)		2740(2397–3125)	25.2(22.05–28.74)		8341(7634–9049)	52.27(47.83–56.7)		4.21(4.19–4.23)	4.02(4.01–4.03)
Guyana	35(25–46)	8.22(5.89–10.99)		150(129–173)	28(24.04–32.27)		342(311–373)	53.15(48.35–57.94)		3.9(3.86–3.95)	3.57(3.55–3.59)
Haiti	105(75–144)	2.97(2.12–4.08)		730(573–912)	10.23(8.03–12.79)		1773(1605–1940)	18.67(16.9–20.44)		3.95(3.91–3.99)	3.33(3.32–3.33)
Honduras	179(127–244)	7.63(5.43–10.39)		1867(1472–2324)	28.54(22.5–35.53)		5549(4975–6124)	59.01(52.9–65.12)		4.21(4.18–4.23)	3.95(3.93–3.96)
Jamaica	131(107–158)	9.55(7.78–11.5)		733(650–818)	34.18(30.32–38.11)		1346(1230–1463)	61.14(55.83–66.44)		4.08(4.04–4.12)	3.19(3.19–3.2)
Mexico	5564(4954–6245)	12.5(11.13–14.03)		32248(30792–33767)	36.09(34.46–37.79)		71022(63286–78758)	64.21(57.21–71.2)		3.36(3.33–3.39)	3.12(3.1–3.15)
Nicaragua	209(159–269)	10.75(8.17–13.85)		1409(1137–1701)	32.42(26.16–39.13)		3381(3085–3676)	56.93(51.96–61.91)		3.51(3.49–3.53)	3.04(3.03–3.05)
Panama	141(109–177)	10(7.73–12.59)		1102(1000–1210)	36.08(32.76–39.64)		2773(2538–3009)	69.2(63.32–75.08)		4.08(4.06–4.11)	3.6(3.58–3.61)
Paraguay	218(157–291)	10.34(7.42–13.75)		1429(1169–1699)	32.08(26.24–38.15)		4031(3706–4355)	69.55(63.95–75.15)		3.6(3.59–3.61)	4.26(4.24–4.29)
Peru	868(709–1050)	7.33(5.99–8.87)		6373(6049–6706)	27.18(25.8–28.6)		25895(23223–28568)	87.47(78.45–96.5)		4.19(4.17–4.22)	6.5(6.46–6.54)
Puerto Rico	413(323–513)	17.35(13.57–21.54)		1119(941–1297)	41.01(34.49–47.56)		1490(1381–1600)	58.6(54.31–62.89)		2.73(2.71–2.75)	2.01(2–2.03)
Saint Kitts and Nevis	4(3–6)	18.14(13.48–23.4)		17(14–20)	46.62(38.73–54.54)		25(22–27)	66.26(59.43–73.09)		3(2.98–3.01)	1.95(1.92–1.98)
Saint Lucia	7(6–9)	9.57(7.66–11.67)		47(43–52)	33.93(30.7–37.29)		86(77–95)	58.49(52.41–64.58)		4.04(4.01–4.06)	3.07(3.04–3.09)

Saint Vincent and the Grenadines	7(5–9)	11.33(8.11–15.32)		25(22–29)	33.57(28.93–38.39)		40(36–44)	54.61(49.09–60.12)		3.45(3.43–3.47)	2.74(2.72–2.77)
Suriname	21(14–30)	9.13(5.92–13.15)		124(99–151)	28.98(23.17–35.42)		257(235–278)	49.21(45.05–53.37)		3.68(3.65–3.71)	3(2.98–3.01)
Trinidad and Tobago	108(87–131)	14.18(11.47–17.26)		340(285–396)	29.15(24.45–33.94)		474(439–508)	38.91(36.08–41.74)		2.27(2.24–2.29)	1.57(1.56–1.58)
United States of America	34953(33179–36764)	18.52(17.58–19.48)		114432(107359–121666)	42.87(40.22–45.58)		177341(162279–192402)	59.23(54.2–64.26)		2.66(2.62–2.7)	1.72(1.71–1.74)
Uruguay	280(219–353)	13.02(10.17–16.39)		902(766–1040)	34.67(29.43–39.97)		1967(1813–2120)	71.27(65.71–76.84)		3.11(3.09–3.12)	4.07(4.03–4.11)
Venezuela (Bolivarian Republic of)	1334(1028–1708)	12.12(9.34–15.52)		4341(3786–4907)	22.84(19.92–25.82)		4922(4509–5335)	21.63(19.81–23.44)		1.98(1.94–2.03)	-0.28(-0.3–-0.27)
**Europe**											
Albania	193(146–248)	9.6(7.22–12.33)		595(520–671)	26.58(23.24–29.98)		1083(1008–1158)	51.77(48.2–55.34)		3.24(3.21–3.26)	3.85(3.83–3.86)
Andorra	4(3–6)	10.84(8.15–13.99)		14(10–18)	20.47(14.71–26.98)		21(19–23)	28.04(25.07–31.01)		2.01(1.97–2.05)	1.8(1.78–1.82)
Armenia	251(199–311)	10.92(8.66–13.52)		614(535–696)	27.87(24.28–31.6)		946(872–1019)	43.49(40.13–46.85)		2.98(2.95–3.02)	2.58(2.54–2.61)
Austria	601(520–686)	9.95(8.61–11.36)		1278(1050–1528)	17.04(14–20.36)		1793(1627–1958)	23.54(21.37–25.72)		1.69(1.66–1.72)	1.79(1.77–1.8)
Azerbaijan	456(359–564)	10.39(8.18–12.86)		2064(1805–2328)	27.5(24.06–31.02)		4012(3713–4311)	45.34(41.96–48.72)		3.09(3.08–3.1)	2.8(2.78–2.82)
Belarus	1333(985–1725)	18.02(13.32–23.32)		1949(1752–2150)	26.55(23.87–29.28)		2020(1880–2161)	28.99(26.98–31.01)		1.22(1.2–1.24)	0.47(0.46–0.48)
Belgium	1032(952–1117)	13.27(12.24–14.37)		2050(1763–2359)	22(18.92–25.31)		3219(2950–3488)	32.28(29.58–34.97)		1.59(1.56–1.61)	2.16(2.13–2.19)
Bosnia and Herzegovina	374(283–478)	11.94(9.04–15.25)		682(535–837)	25.47(19.97–31.27)		986(920–1052)	41.98(39.18–44.78)		2.44(2.34–2.55)	2.68(2.66–2.71)
Bulgaria	857(677–1065)	12.91(10.2–16.04)		1367(1220–1521)	24.25(21.65–26.99)		1662(1542–1781)	33.73(31.31–36.16)		2(1.97–2.02)	1.92(1.9–1.93)
Croatia	479(398–568)	13.04(10.83–15.44)		1150(1040–1258)	35.65(32.24–38.98)		2021(1844–2197)	68.37(62.41–74.33)		3.19(3.17–3.22)	3.7(3.63–3.77)
Cyprus	82(65–100)	14.68(11.73–17.94)		271(202–348)	25.11(18.7–32.25)		419(383–456)	33.83(30.86–36.8)		1.69(1.68–1.71)	1.7(1.69–1.71)
Czechia	1646(1535–1762)	21.78(20.31–23.32)		2706(2447–2976)	31.3(28.31–34.42)		3968(3734–4202)	46.39(43.65–49.12)		1.15(1.11–1.2)	2.27(2.24–2.31)
Denmark	347(313–384)	8.58(7.74–9.48)		679(579–790)	14.3(12.2–16.63)		934(843–1026)	18.56(16.75–20.38)		1.61(1.58–1.64)	1.47(1.45–1.5)
Estonia	172(148–197)	14.9(12.78–17.06)		290(244–338)	26.67(22.41–31.1)		325(298–351)	30.71(28.2–33.22)		1.86(1.82–1.89)	0.78(0.76–0.8)
Finland	573(528–619)	14.9(13.73–16.1)		1075(944–1209)	23.7(20.82–26.66)		1651(1492–1810)	35.48(32.06–38.9)		1.47(1.43–1.51)	2.32(2.26–2.38)
France	4854(4213–5538)	11.29(9.8–12.88)		5736(5037–6492)	10.92(9.59–12.36)		4750(4258–5242)	8.51(7.63–9.39)		-0.1(-0.13–-0.07)	-1.29(-1.32–-1.26)
Georgia	532(399–683)	13.96(10.47–17.93)		1113(983–1241)	38.94(34.38–43.41)		1817(1688–1946)	60.52(56.22–64.83)		3.26(3.23–3.29)	2.46(2.44–2.49)
Germany	9889(9315–10488)	15.35(14.46–16.28)		16940(14535–19366)	24.16(20.73–27.62)		17917(16222–19612)	26.27(23.78–28.76)		1.43(1.39–1.47)	0.53(0.5–0.55)
Greece	1305(1074–1548)	16.72(13.76–19.83)		2914(2605–3234)	33.68(30.11–37.38)		3761(3424–4098)	46.84(42.64–51.03)		2.22(2.19–2.25)	1.9(1.83–1.97)
Greenland	5(4–6)	12.13(9.14–15.5)		12(10–13)	28.17(24.49–31.94)		17(15–19)	40.82(36.47–45.16)		2.66(2.61–2.71)	2.1(2.08–2.12)

Hungary	1136(1015–1263)	14.6(13.04–16.23)		2903(2638–3162)	36.4(33.08–39.65)		4493(4188–4798)	59.25(55.22–63.27)		2.91(2.88–2.93)	2.69(2.68–2.7)
Iceland	18(16–20)	9.84(8.7–11.12)		67(53–82)	22.59(17.95–27.66)		115(105–126)	32.84(29.91–35.78)		2.63(2.61–2.66)	2.13(2.11–2.16)
Ireland	312(260–369)	13.25(11.02–15.66)		1207(1044–1376)	30.84(26.69–35.17)		1988(1838–2138)	41.8(38.64–44.95)		2.67(2.6–2.74)	1.64(1.6–1.68)
Israel	416(371–463)	14.53(12.97–16.18)		1435(1247–1636)	23.39(20.32–26.66)		2363(2147–2579)	28.53(25.92–31.15)		1.5(1.48–1.52)	1.09(1.06–1.11)
Italy	6171(5783–6577)	13.69(12.83–14.59)		10884(9627–12183)	21.55(19.06–24.12)		13378(12112–14645)	27.72(25.1–30.35)		1.43(1.39–1.46)	1.53(1.5–1.56)
Kazakhstan	1177(956–1421)	10.92(8.87–13.19)		2553(2204–2945)	19.34(16.7–22.31)		4849(4507–5191)	28.38(26.38–30.38)		1.8(1.78–1.82)	2.15(2.14–2.17)
Kyrgyzstan	210(173–248)	8.31(6.87–9.83)		1054(882–1232)	24.41(20.42–28.53)		2361(2131–2591)	39.06(35.25–42.87)		3.43(3.41–3.44)	2.37(2.36–2.39)
Latvia	331(269–398)	16.72(13.59–20.11)		454(366–546)	29.81(24.05–35.86)		537(499–576)	38.18(35.43–40.93)		1.83(1.81–1.85)	1.44(1.42–1.47)
Lithuania	578(529–629)	21.41(19.58–23.28)		721(571–872)	31.14(24.67–37.67)		834(777–891)	38.99(36.31–41.67)		1.18(1.17–1.2)	1.36(1.33–1.39)
Luxembourg	41(32–53)	13.69(10.57–17.34)		107(87–128)	20.21(16.49–24.18)		150(138–163)	23.81(21.8–25.83)		1.23(1.18–1.28)	0.93(0.9–0.96)
Malta	63(54–73)	23.9(20.44–27.5)		154(132–178)	34.62(29.54–39.86)		213(196–230)	44.29(40.74–47.84)		1.16(1.14–1.19)	1.35(1.32–1.37)
Montenegro	40(26–58)	9.36(6.12–13.39)		101(72–132)	21.05(15.02–27.5)		156(143–168)	33.25(30.63–35.88)		2.57(2.55–2.58)	2.5(2.46–2.55)
Netherlands (Kingdom of the)	1017(891–1152)	8.72(7.64–9.87)		2462(1989–2985)	16.91(13.66–20.5)		3308(3008–3609)	21.03(19.12–22.94)		2.08(2.05–2.11)	1.29(1.27–1.31)
North Macedonia	201(145–268)	14.37(10.39–19.2)		448(332–576)	30.65(22.72–39.37)		726(676–777)	52.56(48.93–56.19)		2.39(2.37–2.42)	2.92(2.89–2.95)
Norway	287(254–322)	8.83(7.82–9.89)		859(715–1017)	19.77(16.45–23.4)		1377(1258–1496)	28.28(25.83–30.73)		2.55(2.52–2.59)	2(1.98–2.02)
Poland	3938(3569–4329)	14.71(13.33–16.17)		9850(8790–10900)	31.41(28.03–34.76)		17425(15801–19049)	57.32(51.98–62.66)		2.41(2.4–2.43)	3.35(3.31–3.39)
Portugal	963(796–1142)	12.96(10.71–15.36)		2368(2000–2743)	27.06(22.85–31.34)		3395(3113–3676)	39.96(36.65–43.27)		2.33(2.31–2.35)	2.18(2.16–2.19)
Republic of Moldova	568(455–694)	18.93(15.16–23.13)		595(535–658)	25.65(23.06–28.38)		594(549–639)	28.36(26.2–30.52)		1.01(0.97–1.05)	0.17(0.14–0.21)
Romania	1487(1235–1755)	9.04(7.51–10.67)		5906(5284–6520)	38.24(34.21–42.21)		15089(13185–16993)	106(92.62–119.38)		4.64(4.58–4.71)	5.73(5.64–5.82)
Russian Federation	19473(18343–20602)	17.93(16.89–18.97)		32337(28394–36487)	28.05(24.63–31.65)		41207(38012–44402)	35.79(33.02–38.57)		1.42(1.41–1.43)	1.3(1.27–1.33)
Serbia	875(762–995)	14.5(12.63–16.49)		1462(1231–1694)	26.05(21.93–30.18)		2302(2110–2494)	45.79(41.96–49.61)		1.85(1.84–1.86)	3.2(3.15–3.25)
Slovakia	487(420–560)	13.29(11.46–15.29)		1343(1122–1571)	30.28(25.3–35.42)		2518(2322–2715)	57.5(53.02–61.98)		2.62(2.59–2.64)	3.55(3.51–3.59)
Slovenia	184(160–209)	12.3(10.69–13.98)		385(341–430)	22.2(19.66–24.79)		664(594–733)	38.24(34.24–42.23)		1.86(1.83–1.89)	3.13(3.1–3.16)
Spain	4641(4312–4979)	15.81(14.69–16.96)		7634(6491–8793)	19.17(16.3–22.08)		6092(5399–6785)	15.05(13.34–16.76)		0.61(0.59–0.64)	-1.24(-1.28–-1.21)
Sweden	637(578–699)	9.53(8.64–10.45)		1361(1112–1646)	16.41(13.41–19.85)		1833(1672–1994)	19.87(18.12–21.61)		1.72(1.66–1.78)	1.06(1.03–1.08)
Switzerland	538(462–619)	10.11(8.68–11.63)		991(835–1158)	13.74(11.58–16.05)		1317(1205–1430)	16.86(15.42–18.3)		0.96(0.93–0.99)	1.15(1.13–1.17)
Tajikistan	165(114–231)	6.1(4.2–8.52)		1234(1058–1429)	20.81(17.84–24.11)		3699(3419–3979)	41.51(38.37–44.66)		3.91(3.9–3.93)	3.83(3.8–3.86)
Turkey	5231(4788–5687)	16.18(14.81–17.59)		21916(20106–23784)	34.26(31.43–37.18)		35396(32624–38167)	48.19(44.41–51.96)		2.37(2.36–2.39)	1.84(1.83–1.85)
Turkmenistan	139(100–186)	7.03(5.06–9.42)		935(815–1062)	20.16(17.58–22.9)		1990(1846–2134)	31.18(28.92–33.44)		3.36(3.34–3.37)	2.5(2.48–2.52)

Ukraine	7115(5509–8867)	18.39(14.24–22.92)		9847(8633–11075)	29.2(25.6–32.84)		11008(10250–11766)	35.52(33.08–37.97)		1.44(1.42–1.46)	1.25(1.15–1.35)
United Kingdom	5479(5188–5757)	12.42(11.76–13.05)		15442(14361–16512)	28.71(26.7–30.7)		21153(19301–23006)	34.92(31.87–37.98)		2.65(2.62–2.67)	1.08(1.06–1.09)
Uzbekistan	724(563–911)	6.57(5.11–8.27)		6468(5851–7126)	28.52(25.8–31.42)		19348(17720–20975)	63.57(58.22–68.91)		4.7(4.68–4.73)	4.5(4.49–4.52)
**Eastern Mediterranean**											
Afghanistan	92(51–150)	1.68(0.93–2.73)		3529(2981–4096)	17.59(14.86–20.42)		34252(29431–39072)	94.85(81.5–108.2)		7.62(7.59–7.65)	9.44(9.39–9.49)
Bahrain	52(44–62)	16.38(13.71–19.37)		442(358–530)	37.19(30.13–44.53)		788(712–865)	50.53(45.64–55.42)		2.59(2.53–2.65)	1.69(1.64–1.73)
Djibouti	6(2–11)	1.97(0.82–3.93)		79(43–127)	10.87(5.86–17.45)		324(291–357)	33.01(29.63–36.38)		5.48(5.46–5.5)	6.39(6.35–6.43)
Egypt	5480(4810–6206)	17.74(15.57–20.09)		29817(27514–32224)	42.99(39.67–46.46)		61934(56473–67395)	62.12(56.64–67.6)		2.8(2.79–2.82)	2(1.99–2.01)
Iran (Islamic Republic of)	2829(2359–3336)	9.64(8.04–11.37)		16428(15381–17540)	25.26(23.65–26.97)		32734(29722–35746)	40.64(36.9–44.38)		3.07(3.05–3.1)	2.55(2.54–2.56)
Iraq	1443(1138–1769)	17.21(13.58–21.1)		9233(8153–10419)	37.36(32.99–42.16)		23137(21215–25058)	57.75(52.95–62.54)		2.45(2.44–2.47)	2.35(2.32–2.37)
Jordan	331(288–376)	19.05(16.59–21.65)		2497(2302–2698)	35.61(32.84–38.48)		4563(4166–4960)	44.65(40.77–48.54)		1.99(1.95–2.03)	1.13(1.11–1.15)
Kuwait	270(241–298)	27.47(24.54–30.37)		1609(1439–1779)	45.36(40.55–50.14)		2194(1984–2404)	47.34(42.8–51.88)		1.57(1.47–1.66)	0.47(0.42–0.53)
Lebanon	261(214–313)	12.71(10.41–15.27)		1203(1051–1363)	31.07(27.15–35.22)		2137(1960–2314)	43.34(39.76–46.93)		2.83(2.8–2.85)	1.96(1.93–1.98)
Libya	357(280–440)	15.72(12.34–19.4)		1721(1445–2004)	36.15(30.36–42.08)		3275(3032–3518)	50.44(46.7–54.18)		2.65(2.61–2.68)	1.79(1.77–1.8)
Morocco	930(759–1127)	7.2(5.88–8.73)		5663(4877–6525)	22.13(19.06–25.5)		12346(11362–13329)	38.88(35.78–41.97)		3.58(3.56–3.6)	3.11(3.11–3.12)
State of Palestine	156(132–182)	16.59(14.03–19.3)		958(884–1032)	32.82(30.28–35.36)		2109(1919–2299)	44.98(40.93–49.03)		2.15(2.14–2.17)	1.6(1.58–1.63)
Oman	120(105–135)	13.43(11.82–15.16)		1004(878–1143)	30.19(26.39–34.38)		2686(2396–2976)	51.45(45.9–57.01)		2.57(2.5–2.64)	2.82(2.81–2.83)
Pakistan	1591(1273–1956)	2.75(2.2–3.38)		29986(26651–33554)	21.85(19.42–24.45)		187998(166916–209079)	92.2(81.86–102.54)		6.69(6.67–6.71)	8.08(8.03–8.13)
Qatar	54(42–69)	17.95(13.92–22.75)		1045(882–1211)	43.76(36.93–50.7)		2034(1783–2284)	67.57(59.25–75.88)		2.84(2.79–2.88)	2.3(2.24–2.36)
Saudi Arabia	1019(958–1083)	18.54(17.42–19.7)		9405(8254–10618)	41.11(36.08–46.41)		18602(16831–20373)	58.67(53.09–64.26)		2.5(2.43–2.56)	2.04(2–2.07)
Somalia	88(44–157)	2.66(1.34–4.73)		1049(726–1430)	12.7(8.79–17.31)		6013(5419–6607)	38.82(34.99–42.66)		5.01(4.98–5.03)	6.33(6.28–6.37)
Sudan	354(225–524)	3.39(2.16–5.02)		4010(3306–4760)	15.5(12.78–18.4)		22131(19636–24626)	52.7(46.76–58.64)		4.87(4.84–4.9)	6.95(6.9–7.01)
Syrian Arab Republic	860(691–1048)	14.73(11.83–17.94)		4259(3381–5178)	31.18(24.75–37.91)		11251(10315–12188)	48.63(44.58–52.67)		2.37(2.31–2.43)	2.54(2.5–2.58)
Tunisia	490(405–583)	10.52(8.7–12.52)		2433(1962–2943)	28.26(22.79–34.19)		4594(4222–4967)	44.55(40.94–48.16)		3.14(3.13–3.16)	2.5(2.48–2.53)
United Arab Emirates	224(186–267)	17.99(14.9–21.43)		2617(2273–2976)	31.55(27.4–35.88)		4069(3570–4568)	37.21(32.65–41.77)		1.78(1.74–1.83)	0.82(0.77–0.88)
Yemen	177(129–236)	3(2.19–4.01)		2309(1746–3007)	11.56(8.74–15.05)		10386(9400–11373)	30.01(27.16–32.86)		4.32(4.28–4.35)	5.16(5.16–5.17)
**South-East Asia**											

Bangladesh	170(113–238)	0.3(0.2–0.42)		5833(4940–6837)	5.29(4.48–6.2)		49413(42372–56453)	33.73(28.92–38.53)		9.43(9.33–9.53)	10.35(10.32–10.38)
Bhutan	5(3–8)	1.54(0.87–2.56)		68(57–80)	11.98(10.08–14.03)		286(254–317)	41.29(36.76–45.83)		6.6(6.58–6.63)	7.1(7.08–7.12)
Democratic People's Republic of Korea	285(113–581)	2(0.79–4.08)		2210(1155–3593)	10.87(5.68–17.67)		7026(6379–7674)	33.02(29.98–36.07)		5.43(5.4–5.47)	6.18(6.16–6.21)
India	3342(2865–3915)	0.7(0.6–0.82)		71027(63540–79007)	7.21(6.45–8.02)		366349(322887–409810)	29.73(26.21–33.26)		7.56(7.49–7.63)	7.96(7.93–7.99)
Indonesia	1468(1120–1880)	1.39(1.06–1.78)		22362(18959–26095)	11.5(9.75–13.42)		109869(96593–123144)	46.78(41.13–52.44)		6.85(6.79–6.9)	7.89(7.85–7.94)
Maldives	3(2–4)	3.04(2.1–4.22)		70(62–79)	17.57(15.43–19.82)		230(203–257)	48.4(42.68–54.12)		5.64(5.62–5.66)	5.93(5.91–5.95)
Myanmar	407(254–626)	1.78(1.11–2.74)		2851(2189–3603)	7.54(5.79–9.53)		7576(6880–8272)	17.35(15.76–18.95)		4.6(4.58–4.62)	4.6(4.6–4.61)
Nepal	45(29–68)	0.44(0.28–0.67)		1269(1059–1496)	6.58(5.49–7.76)		10245(8734–11757)	42.71(36.41–49.02)		8.83(8.76–8.9)	10.51(10.47–10.55)
Sri Lanka	106(68–158)	1.06(0.68–1.58)		1758(1494–2044)	10.61(9.02–12.34)		7254(6608–7901)	38.11(34.71–41.5)		7.47(7.44–7.51)	7.19(7.16–7.23)
Thailand	1113(929–1326)	3.2(2.67–3.81)		8424(7338–9602)	14.51(12.64–16.54)		26123(22752–29495)	43.87(38.21–49.53)		4.83(4.82–4.85)	6.01(5.97–6.06)
Timor-Leste	1(1–2)	0.26(0.13–0.45)		18(13–25)	2.25(1.58–3.09)		139(120–159)	11.77(10.11–13.44)		6.96(6.87–7.06)	9.65(9.62–9.68)
**Western Pacific**											
American Samoa	16(15–16)	61.03(58.63–63.46)		24(22–26)	75.56(68.79–81.48)		26(23–28)	86.47(78.44–94.51)		0.67(0.66–0.68)	0.79(0.77–0.81)
Australia	1610(1501–1727)	12.84(11.97–13.77)		6518(5932–7138)	31.82(28.96–34.85)		11580(10620–12539)	47.2(43.29–51.11)		2.86(2.81–2.91)	2.13(2.11–2.14)
Brunei Darussalam	11(8–16)	7.45(4.98–10.49)		110(95–125)	32.38(28.05–36.77)		243(217–268)	60.14(53.86–66.42)		4.7(4.66–4.74)	3.46(3.44–3.48)
Cambodia	16(10–26)	0.44(0.27–0.69)		482(403–573)	4.39(3.67–5.22)		2866(2573–3160)	19.83(17.8–21.86)		7.43(7.33–7.52)	8.58(8.54–8.61)
China	8556(7805–9381)	1.14(1.04–1.25)		92968(81418–105425)	8.21(7.19–9.31)		410796(360014–461577)	34.5(30.24–38.77)		6.38(6.33–6.42)	8.01(7.95–8.07)
Cook Islands	4(4–5)	45.25(39.65–51.27)		7(6–8)	68.42(62.54–73.83)		7(6–8)	83.48(74.07–92.88)		1.3(1.29–1.31)	1.12(1.08–1.16)
Fiji	74(63–85)	17.2(14.74–19.7)		208(173–246)	33.84(28.02–39.95)		415(382–449)	58.54(53.78–63.31)		2.14(2.13–2.15)	3.03(3–3.06)
French Polynesia	39(32–46)	33.22(27.12–39.77)		102(87–117)	48.42(41.33–55.57)		148(138–159)	61.61(57.16–66.06)		1.18(1.16–1.19)	1.34(1.31–1.37)
Japan	1861(1728–2004)	1.96(1.82–2.11)		5276(4411–6195)	4.94(4.13–5.8)		9136(8377–9894)	9.31(8.54–10.09)		2.92(2.87–2.96)	3.55(3.54–3.56)
Kiribati	12(11–14)	30.04(26.55–33.73)		36(32–40)	46.19(41.01–51.42)		59(53–65)	55.17(49.77–60.57)		1.34(1.3–1.39)	0.97(0.96–0.99)
Lao People's Democratic Republic	26(15–41)	1.21(0.69–1.93)		371(264–500)	7.76(5.53–10.45)		1741(1580–1903)	26.8(24.31–29.28)		5.99(5.95–6.03)	7.03(7.01–7.05)
Malaysia	486(385–603)	4.82(3.81–5.97)		5620(4985–6274)	22.4(19.87–25.01)		15990(14238–17742)	48.41(43.11–53.72)		4.9(4.87–4.94)	4.23(4.22–4.24)
Marshall Islands	6(5–7)	31.05(26.4–35.87)		11(10–12)	47.29(42.98–51.46)		10(8–11)	53.5(47.23–59.78)		1.33(1.3–1.36)	0.86(0.81–0.9)

Micronesia (Federated States of)	15(12–17)	30.96(26.1–36.15)		31(27–35)	45.6(39.73–51.31)		50(45–54)	61.41(55.81–67.01)		1.22(1.2–1.24)	1.68(1.65–1.7)
Mongolia	69(52–88)	6.1(4.61–7.81)		510(451–571)	23.99(21.24–26.86)		1320(1210–1431)	44.47(40.74–48.19)		4.38(4.35–4.42)	3.39(3.35–3.43)
Nauru	4(3–4)	67.87(65.66–70.09)		5(4–5)	70.18(65.06–74.97)		7(6–7)	74.18(65.02–83.35)		0.1(0.08–0.12)	0.27(0.26–0.28)
New Zealand	321(290–355)	13.2(11.91–14.59)		1364(1287–1442)	34.25(32.31–36.21)		2180(1990–2369)	47.8(43.64–51.96)		3.02(2.99–3.04)	1.8(1.78–1.81)
Niue	1(1–1)	47.53(40.69–54.52)		1(1–1)	66.46(60.02–72.4)		1(1–1)	84.98(71.19–98.78)		1.05(1.03–1.08)	1.45(1.4–1.5)
Palau	3(3–4)	32.09(25.88–38.65)		6(5–6)	42.2(37.27–47.22)		6(6–7)	47.67(42.54–52.81)		0.86(0.84–0.87)	0.63(0.62–0.65)
Papua New Guinea	231(187–281)	11.57(9.33–14.07)		1228(900–1607)	20.12(14.75–26.34)		2952(2711–3193)	33.3(30.58–36.01)		1.74(1.73–1.75)	2.7(2.69–2.71)
Philippines	519(432–617)	1.6(1.33–1.9)		6454(5095–7946)	8.74(6.9–10.76)		25381(22353–28409)	26.2(23.08–29.33)		5.44(5.4–5.47)	6.06(6.03–6.09)
Republic of Korea	429(348–522)	1.43(1.16–1.74)		2997(2681–3335)	6.73(6.02–7.49)		9476(8634–10318)	21.34(19.45–23.24)		4.96(4.93–4.98)	6.53(6.5–6.56)
Samoa	29(27–31)	33.56(31.6–35.58)		73(66–79)	61.24(55.73–66.53)		137(123–150)	90.06(81.23–98.89)		1.9(1.88–1.91)	2.1(2.02–2.17)
Singapore	87(74–100)	3.86(3.31–4.47)		645(531–778)	13.54(11.15–16.32)		2234(2014–2453)	41.97(37.85–46.09)		4(3.95–4.06)	6.32(6.31–6.32)
Solomon Islands	18(14–24)	11.74(8.7–15.43)		95(77–113)	21.63(17.71–25.8)		256(233–279)	35.89(32.67–39.11)		1.92(1.91–1.94)	2.83(2.8–2.86)
Tokelau	1(0–1)	52.18(46.06–58.24)		1(1–1)	69.21(63.31–74.7)		2(2–3)	88.82(74.73–102.92)		0.88(0.85–0.91)	1.39(1.37–1.41)
Tonga	23(20–26)	43.84(38.45–49.3)		43(40–45)	70.54(66.37–74.48)		69(62–76)	105.24(94.95–115.54)		1.5(1.48–1.51)	2.22(2.19–2.25)
Tuvalu	2(2–3)	46.24(38.76–53.67)		4(4–4)	63.93(58.32–69.25)		5(4–6)	80.42(70.18–90.65)		1.01(1–1.03)	1.28(1.24–1.32)
Vanuatu	9(8–11)	12.68(10.33–15.34)		35(27–43)	19.95(15.34–25.04)		78(70–85)	27.92(25.31–30.53)		1.42(1.41–1.43)	1.81(1.79–1.82)
Viet Nam	72(54–90)	0.2(0.15–0.25)		1489(1131–1918)	2.08(1.58–2.68)		12467(10105–14828)	14.6(11.83–17.37)		7.64(7.41–7.88)	11.04(10.94–11.14)
